# Glutamate receptor–T cell receptor signaling potentiates full CD8^+^ T cell activation and effector function in tumor immunity

**DOI:** 10.1016/j.isci.2025.112772

**Published:** 2025-08-11

**Authors:** Maria Teresa P. de Aquino, Thomas W. Hodo, Salvador González Ochoa, Roman V. Uzhachenko, Muna A. Mohammed, J. Shawn Goodwin, Thanigaivelan Kanagasabai, Alla V. Ivanova, Anil Shanker

**Affiliations:** 1 Department of Biochemistry, Cancer Biology, Neuroscience and Pharmacology, School of Medicine, Meharry Medical College, Nashville, TN 37208, USA; 2 Department of Biomedical Sciences, School of Graduate Studies, Meharry Medical College, Nashville, TN 37208, USA; 3 Host-Tumor Interactions Research Program, Vanderbilt-Ingram Comprehensive Cancer Center, Vanderbilt University School of Medicine, Nashville, TN 37235, USA

**Keywords:** immune response, immunology

## Abstract

Glutamate is best known as an excitatory neurotransmitter. However, its roles in T cell immunity remain underrecognized. We investigated the interplay between glutamate receptors (GluRs) and T cell receptors (TCRs) during CD8^+^ T cell activation. Our findings reveal that GluR expression in CD8^+^ T lymphocytes strongly correlates with the activation of TCR-CD28 signaling, enhancing their antitumor effector responses. Conversely, pharmacologic antagonism of GluRs in activated CD8^+^ T cells disrupts the colocalization of GluR with TCRVβ8.1, reduces the phosphorylation of TCR-signaling intermediates, alters calcium flux, and impairs the metabolic switch to glycolysis essential for T cell activation. Moreover, these disruptions blunt clonal proliferation and compromise the tumor-cytolytic capacity of CD8^+^ T cells. Thus, the glutamatergic system—*via* the GluR−TCR signaling complex—plays a critical amplifier role in activating CD8^+^ T cells and eliciting their full antitumor activity. This mechanistic insight reveals a previously underappreciated axis in T cell biology and opens avenues for immunotherapy regimens targeting GluR−TCR interactions to augment T cell–mediated responses in cancer and potentially other immunopathologies.

**Video Abstract:**

## Highlights

• Expression of GluR, CD69 and CD25 is critical for the activation of CD8^+^ T lymphocytes
• Inhibiting GluR signaling blunts CD8^+^ T cell proliferation, glycolysis and effector function
• Activation of the GluR−TCR−CD28 axis elicits full CD8^+^ T cell antitumor effector responses
• Engagement of mGluR1 shapes T cell immunity in the solid tumor microenvironment

## Graphical abstract

**Figure:**
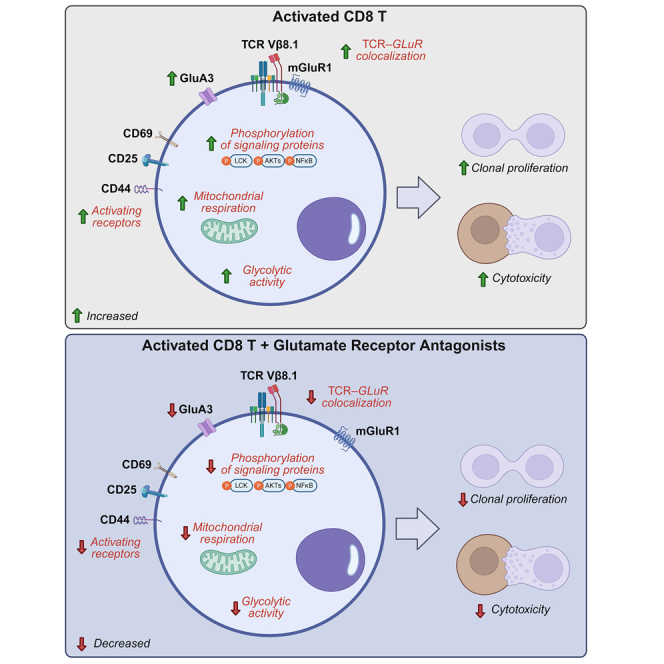

## Teaser

Immune response; Cancer immunology

## Introduction

The glutamate receptor (GluR) family comprises a diverse range of receptors, categorized as either ionotropic (iGluR) or metabotropic (mGluR). Ionotropic receptors form ion channel pores in the cell membrane, which open upon ligand binding to enable the passage of ions such as Na^+^, K^+^, Cl^−^, or Ca^2+^ into the cell. These iGluR receptors are subdivided into N-methyl-D-aspartate (NMDA) and non-NMDA receptors. NMDA receptors bind to glutamate and other ligands and are more permeable to Ca^2+^ ions. In contrast, non-NMDA receptors, such as AMPA (GluA1–4) and Kainate (KA; GluK1–5), exclusively bind to glutamate and are more permeable to Na^+^ or K^+^ ions. Conversely, mGluRs, also known as G protein-coupled receptors, modulate the opening of ion channels through G protein signal transduction mechanisms. Subsequently, these mechanisms initiate a signaling cascade mediated by secondary messengers following the binding of glutamate.^1^^,^^2^

Glutamate and its receptors were previously thought to have functional roles solely within the nervous system. Nonetheless, a wealth of research over the past few decades has revealed that GluRs and glutamate are found in non-neuronal cells, particularly immune cells,^3^^,^^4^ suggesting a dual role of glutamate as a neurotransmitter and an immunomodulator.^1^^,^^5^ Notably, glutamate plays a crucial role in synthesizing amino acids and glutathione, and its concentration in the environment can trigger either pro- or anti-inflammatory responses in immune cells.^1^^,^^2^^,^^4^^,^^6^^,^^7^ Moreover, some studies suggest that the binding of glutamate agonists to GluRs can lead to excitotoxicity, necrotic cell death, or influence T cell fate.^8^^,^^9^^,^^10^^,^^11^

T cell activation is central to the adaptive immune response and begins when a peptide antigen, presented by a major histocompatibility complex (MHC) molecule, engages the T cell receptor (TCR)–CD3 complex. Peptide-MHC–TCR ligation initiates a signaling cascade in which the Src family protein tyrosine kinase LCK phosphorylates immunoreceptor tyrosine-based activation motifs (ITAMs) within the CD3 and TCR ζ chains, creating docking sites that recruit and activate ZAP-70. Activated ZAP-70 phosphorylates LAT, the scaffolding protein that organizes the signalosome at the plasma membrane. The assembled signalosome complex propagates downstream signaling through MAPK/ERK, Janus kinase/STAT, NFAT, and NF-κB pathways.^12^^,^^13^^,^^14^ Collectively, these cascades amplify biochemical transduction of the peptide-MHC–TCR signal, driving clonal expansion and differentiation of activated T cells into specialized cytolytic T cell (CTL) effector subsets with distinct functions.^15^^,^^16^

Nevertheless, studies have challenged the notion that the transmembrane adaptor LAT is the sole factor diversifying signals downstream of TCR. It has been shown that T cell activation and TCR signal propagation are much more diverse and involve multiple distinct signaling pathways.^14^ Signal propagation through the TCR complex occurs through microclusters, submicron-sized TCR aggregates, and associated signaling molecules formed immediately after antigen presentation. These microclusters represent discrete signaling units during T cell activation. In addition, depending on the nature of T cell stimulation, calcium fluxes are detected shortly after microcluster formation,^16^^,^^17^ which triggers cascades of signaling events involving ERK, JNK, NFAT, and NF-κB pathways, leading to an increase in glucose and glutamine uptake by activated T cells, supporting their higher energy demand.^12^^,^^18^ Based on this, it is evident that the molecular architecture of the TCR network is far more intricate and complex than previously thought.

Recent studies indicate that the absence or inhibition of molecules responsible for modulating glutamate processing significantly influences essential metabolic programs within distinct subtypes of T cells, particularly CD4^+^ and CD8^+^ T cells.^19^^,^^20^ However, the precise implications of GluRs interacting with other receptors within TCR microclusters remain undetermined.

This study demonstrates that T cell activation depends on the glutamatergic system of the GluR−TCR complexes and their downstream signaling pathways. In addition, we found that the full effector function of CD8^+^ T cells, including proliferation and cytotoxicity in the tumor microenvironment, is hindered when GluR signaling is disrupted by specific inhibitors. Together, these findings highlight a previously unrecognized mechanistic axis whereby GluR−TCR interactions amplify signaling required for full CD8^+^ T cell effector function.

## Results

### High glutamate receptor expression is linked with T cell receptor activation in tumor-infiltrating and activated lymphocytes

**Figure 1:**
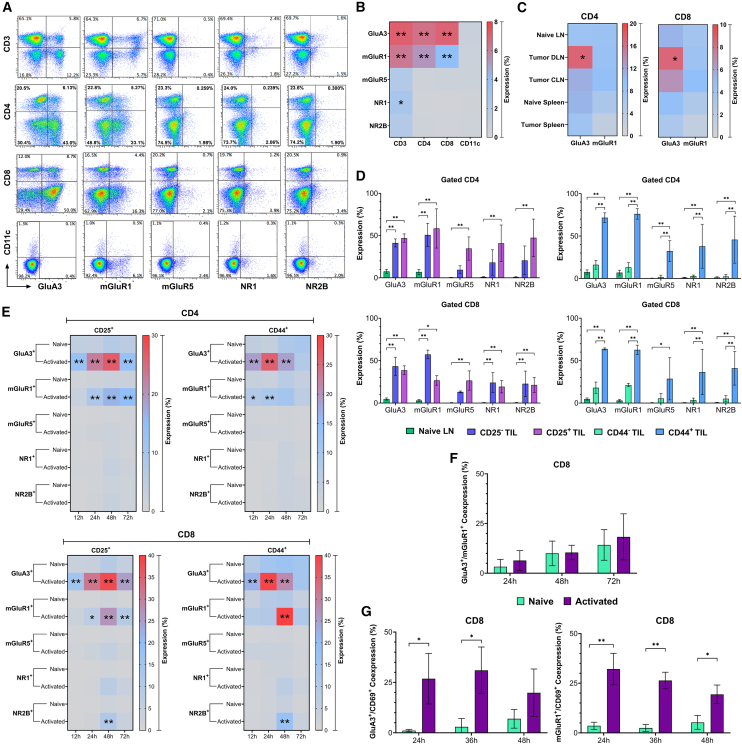
Glutamate receptor expression is high in TCR-activated tumor-infiltrating and activated lymphocytes (A) Panels of dot plots display the frequency of glutamate receptors GluA3, mGluR1, mGluR5, NR1, and NR2B gated on live immune cells CD3^+^, CD4^+^, CD8^+^, and CD11c^+^ from spleen and lymph nodes of tumor-free Balb/c mice. These dot plots are representative of 3 independent experiments. (B) Heatmaps depict the expression levels of GluR on T cells and CD11c^+^ APCs analyzed from the flow cytometry dot plots within the double-positive population (upper right axis). The data presented in the heatmaps reflect the mean values ± standard error of the Mean (SEM), with a sample size of *n* = 3. (C) The heatmaps illustrate the expression levels of GluA3 and mGluR1 in CD4^+^ and CD8^+^ T lymphocytes obtained from both normal and tumor tissues of tumor-bearing mice, analyzed 10 days post-orthotopic injection. The data represented in the heatmaps reflect the Mean ± SEM (*n* = 3) for both conditions. (D) CD4^+^ and CD8^+^ TILs from mammary 4T1.2HA tumor-bearing mice were analyzed for glutamate receptor expression in CD25/CD44 expressing or non-expressing cells. Bars represent the Mean ± SEM (*n* = 3). (E) Heatmaps showing GluRs expression kinetics on CD25^+^CD44^+^ gated CD4^+^ or CD8^+^ activated and naive T cells at various post-*in-vitro* activation time points with anti-CD3 and anti-CD28 (0.5 μg/mL each). Heatmaps represent the Mean ± SEM (*n* = 3). (F) Frequency of double-positive GluA3 and mGluR1 on gated CD8^+^ T cells represented using column graphs. Bars represent the Mean ± SEM (*n* = 3). (G) Bar graphs depicts the double-positive co-expression of GluA3 or mGluR1 with CD69 at various time points. Bars represent the Mean ± SEM (*n* = 3). ^∗^*p* ≤ 0.05, ^∗∗^*p* ≤ 0.01. Two-way ANOVA with multiple comparisons. For a Figure360 author presentation of Figure 1, see https://doi.org/10.1016/j.isci.2025.112772#mmc2.

We examined the baseline differences in the GluR expression levels among subsets of T lymphocytes and antigen-presenting cells (APCs) isolated from combined spleen and lymph nodes of tumor-free mice. Specifically, we assessed their expression on CD3^+^, CD4^+^, CD8^+^ T cells, and CD11c^+^ cells (Figure 1A). CD4^+^ and CD8^+^ T lymphocytes expressed GluA3 (glutamate ionotropic receptor AMPA3) and mGluR1 (glutamate receptor, metabotropic 1) at a moderate level. The expression of mGluR5 (glutamate receptor, metabotropic 5) and ionotropic NMDA receptors NR1 and NR2B were also observed, but at lower levels (Figures 1A and 1B). CD11c^+^ antigen-presenting cells (APCs) showed minimal expression of glutamate receptors (GluRs) compared to T cells, with mGluR5 exhibiting negligible expression across all evaluated cell subtypes. These results suggest that among the analyzed murine immune cell populations, only CD3^+^ T cells significantly express GluRs, while APCs, such as CD11c^+^ DC, express them at minimal levels supporting the idea that the expression of GluRs may have distinct effects on APCs and T cells.^9^^,^^21^^,^^22^

Next, we sought to examine the impact of the tumor environment on the GluR profiles of lymphocytes. We orthotopically administered 4T1.2 mammary adenocarcinoma cells expressing hemagglutinin (4T1.2HA), a low avidity antigen-expressing tumor model.^23^ Ten days post-injection, tumor-infiltrating lymphocytes (TILs) were harvested and analyzed for GluRs expression. We found that T lymphocytes from draining lymph nodes have increased expression of the GluA3 receptor (Figures 1C and 1D). Furthermore, analysis of GluR expression on CD25^+^ and CD44^+^ T cells showed that TILs expressed significantly higher levels of glutamate receptors than naive T-cells (Figure 1D). This suggests that the expression of glutamate receptors is closely related to the activation state of tumor-infiltrated lymphocytes and is associated with memory-like CD4⁺ and CD8⁺ T cell populations.

To confirm the correlation between T cell activation, GluR expression, and development of immune response, CD4^+^ and CD8^+^ T cells were activated *in vitro* through their TCRs using agonistic anti-CD3 and anti-CD28 antibodies.^24^ The presence of GluRs on CD4^+^ and CD8^+^ T cells, specifically CD25^+^ and CD44^+^ cells, was directly related to their activation state (Figure 1E). Furthermore, all analyzed receptors on both T cell subtypes were elevated around 24 h and peaked around 48 h post-TCR activation compared to naive controls (Figure 1E). The receptor levels decreased by 72 h post-activation (Figure 1E), suggesting that GluRs may be involved at early T cell activation stages.^13^

Further analysis of GluA3 and mGluR1 expression on activated CD8^+^ T cells revealed that their individual expression peaked at 48 h post-activation *in vitro* (Figure 1E). Interestingly, the frequency of their co-expression on CD8^+^ T cells remained unchanged between activated and naive cells at all analyzed time points (Figure 1F), suggesting that the co-expression of these GluRs is independent of the activation state. However, we observed an increase in the co-expression of GluA3 and mGluR1 with CD69 on CD8^+^ T cells, underscoring the importance of these GluRs for the activation of CD8^+^CD69^+^ T cells (Figure 1G). Based on these results, our research delved into the implications of GluA3 and mGluR1 molecules in activating CD8^+^ T lymphocytes.

### The modulation of the glutamatergic system is correlated with prolonged T cell receptor activation in CD8^+^ T cells

**Figure 2:**
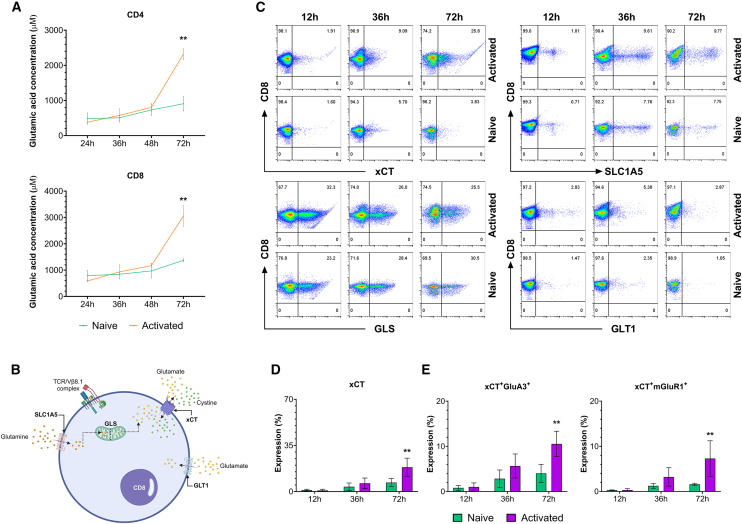
CD8^+^ T cells express molecules of the glutamatergic system (A) Quantification of free glutamic acid from CD4^+^ and CD8^+^ T cell supernatants at specific time points after TCR activation. Time-point line graph represents the Mean ± SEM (*n* = 3). (B) Schematic image representing the molecules of the glutamatergic system displayed on CD8^+^ T cells. SLC1A5 (ASCT2), Glutamine transporter; GLT1, Glutamate transporter; xCT, Glutamate-cystine exchanger; GLS, Glutaminase. (C) Expression of glutamatergic system transporters in naive or activated CD8⁺ T cells at 12, 36, and 72 h post-activation, shown in dot plots. Dot plots are representative of 3 independent experiments. (D and E) Bar graphs depicting xCT protein expression in CD8^+^ T cell subpopulation and co-expression with GluA3 and mGluR1 at 24, 48, and 72 h. Bars represent the Mean ± SEM (*n* = 3). ^∗∗^*p* ≤ 0.01. Two-way ANOVA with multiple comparisons. For a Figure360 author presentation of Figure 2, see https://doi.org/10.1016/j.isci.2025.112772#mmc3.

Following our observation of the correlation between the increased GluR expression and TCR activation, we questioned if T cells can generate endogenous glutamate and elicit responses through self-produced glutamate without the utilization of exogenous glutamate typically provided by APCs.^10^ We measured the concentration of glutamic acid in cultured media collected from CD4^+^ and CD8^+^ T cells at different time points (0–72 h) after TCR activation (Figure 2A). We determined that CD4^+^ and CD8^+^ T cells secreted glutamate at increased levels and reached the maximum release peak around 72 h post-activation. To further understand the glutamatergic system in T cells, we assessed the expression of specific glutamate transport molecules implicated in glutamate signaling and exchange (Figure 2B) at 12, 36, and 72 h post-activation. This analysis encompassed the evaluation of the cystine/glutamate antiporter (xCT), responsible for the release of glutamate in exchange for cystine, the glutamine transporter ASCT2 (SLC1A5), facilitating glutamine transportation into the cellular space, the glial glutamate transporter (GLT1) responsible for sequestering glutamate from the extracellular space, and the enzyme glutaminase (GLS), which catalyzes the conversion of glutamine to glutamate within T cells (Figure 2C).

Interestingly, the transporters, SLC1A5, GLT1, and the enzyme GLS were found to be constitutively expressed in CD8^+^ T cells independently of their activation or naive state (Figure S2A), indicating their importance for maintaining the viability of CD8^+^ T cells. Furthermore, no changes in the expression of these molecules were observed in GluA3^+^ or mGluR1^+^ CD8^+^ T cells (Figure S2B). Conversely, the expression of antiporter xCT increased at 36 h post-T cell activation, and was significantly higher at 72 h compared with naive CD8^+^ T cells (Figure 2D). Additionally, xCT showed a temporal expression pattern in both GluA3^+^ and mGluR1^+^ activated CD8^+^ T-cells (Figure 2E). These findings suggest that the constitutive expression of certain glutamate transporters may facilitate the regulation, production, uptake, synthesis, and release of glutamate in CD8^+^ T cells, independently of their activation state. On the other hand, the increased expression of other transporters, such as xCT, may represent a regulatory mechanism sustaining T cell function following activation.

### Disruption of group I metabotropic receptor signaling impacts T cell receptor-dependent activation

**Figure 3:**
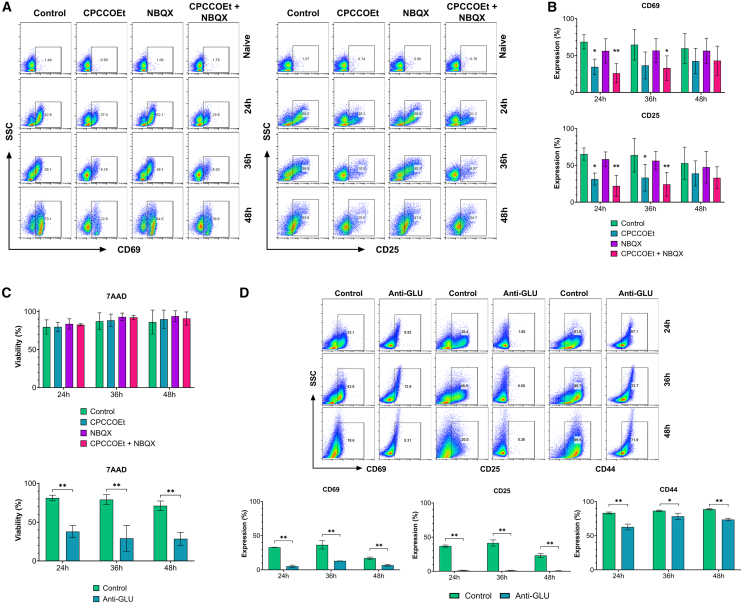
Blockade of GluR signaling on CD8^+^ T cells reduces the expression of activation molecules (A) Dot plots displaying the expression of CD25 or CD69 on naive and activated CD8^+^ T cells with or without treatments of CPCCOEt (non-competitive mGluR1/mGluR5 antagonist; 75 μM), NBQX (competitive AMPA/GluA3 antagonist; 60 μM), or both. These dot plot data are representative of 3 independent experiments. (B) Bar graphs showing the percentage of CD8^+^ T cells expressing CD25 or CD69 at 24, 36, and 48 h after activation with or without GluR antagonist treatments. Bars represent the Mean ± SEM (*n* = 5). (C) Bar graphs depicting the impact of GluR and glutamate inhibitors on CD8^+^ T cell viability. Bars represent the Mean ± SEM (*n* = 3). (D) Decrease of CD8^+^ T cell activation markers CD25, CD69, and CD44 shown in dot plot panels, and graphs after soluble glutamate blockade with anti-glutamate (1:100). These dot plot data are representative of 3 independent experiments. Bars represent the Mean ± SEM (*n* = 3). ^∗^*p* ≤ 0.05, ^∗∗^*p* ≤ 0.01. Two-way ANOVA with multiple comparisons. For a Figure360 author presentation of Figure 3, see https://doi.org/10.1016/j.isci.2025.112772#mmc4.

Our findings that glutamate receptors are prominently expressed on T cells and the ability of these cells to produce and release glutamate suggest a functional role of GluR in the process of T cell activation. To evaluate this, we analyzed the effects of blocking the metabotropic and AMPA ionotropic glutamate receptors on CD8^+^ T cell functions. We treated naive and activated CD8^+^ T cells with two inhibitors of GluR signaling, administered individually or in combination, and analyzed the expression of CD69 and CD25, two pivotal receptors for activation. (Figure 3A). The first inhibitor, CPCCOEt, is a non-competitive antagonist that blocks downstream signaling upon glutamate binding to mGluR1. The second inhibitor, NBQX, is a highly selective competitive antagonist of AMPA and KA ionotropic glutamate receptors such as GluA3, which interferes with glutamate binding.^25^

Activated CD8^+^ T cells treated with the mGluR1 signaling inhibitor or the combination of two inhibitors exhibited significantly lower expression of the activation markers CD25 and CD69 at 24 h and 36 h post-activation compared with activated untreated control cells. At 48h post-activation, no differences in CD25 and CD69 expression were observed between treated and control groups (Figure 3B), suggesting that CD8^+^ T cells can re-establish the expression of CD25 and CD69 after treatment. The expression of molecules involved in adhesion, migration, and generation of T cell memory (CD62L and LFA-1) was not significantly affected by the treatment of GluA3 and mGluR1 inhibitors (Figure S3). In addition, no effects of GluR antagonists were observed on naive CD8^+^ T cells (Figures 3A and 3B).

Glutamate is an essential amino acid critical for T cell proliferation and survival. Therefore, we investigated the effect of blocking the GluR signaling axis on CD8^+^ T cell viability at 24, 36, and 48 h post-treatment. We found no reduction in CD8^+^ T cell viability after inhibition of mGluR1 or GluA3 with their respective antagonists (Figure S4). These findings indicate that the decrease in T cell activation was not due to the absence of glutamate as a survival factor but rather to the lack of essential activation signals mediated through GluR signaling.

We also evaluated the autocrine effect of free glutamate on CD8^+^ T cell activation using an anti-glutamate antibody, which reduces free glutamate availability but not glutamate secretion or production since it does not bind to glutamine, the primary source of glutamate synthesis. Reduction of free glutamate resulted in decreased viability of CD8^+^ T cells despite free access to glutamine available from cell media (Figure 3C), consistent with the essential role of glutamate in cell metabolism. In addition, reduced free glutamate decreased CD25, CD69, and CD44 expression up to 48 h post-activation, similar to the effect of GluR blockade (Figure 3D). Thus, the decreased T cell activation observed after free glutamate sequestration is likely due in part to its effect on cell viability.

Taken together, these data indicate that while both ionotropic and metabotropic GluRs play integral roles in the activation process of cytotoxic CD8^+^ T cells, the inhibition of metabotropic mGluR1 receptor signaling significantly disrupts the expression of molecules essential to TCR-mediated activation.

### Activated CD8^+^ T cells co-express and colocalize mGluR1 and T cell receptor

**Figure 4:**
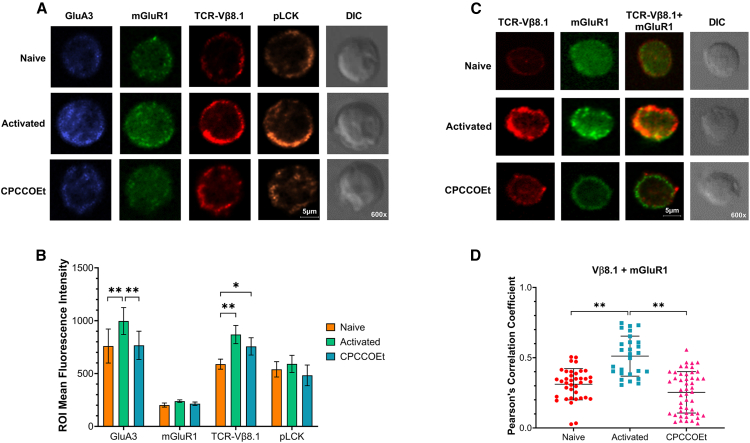
Activated and naive CD8^+^ T cells express GluR, but only activated CD8^+^ T cells exhibit co-occurrence between TCR and GluR (A) Confocal images showing expression of GluA3, mGluR1, TCR Vβ8.1, and phosphorylated Lck (pLck) in naive and activated CD8^+^ T cells at 48 h post-activation. Differential interference contrast (DIC) imaging was also performed. (B) Column graphs displaying the mean fluorescence intensity (MFI) of GluR, Vβ8.1, and pLck in the region of interest (ROI) of naive and activated CD8^+^ T cells treated or untreated with CPCCOEt after 48 h post-activation. Bars represent the Mean ± SEM of 7 cells ROI per independent experiment (*n* = 3). (C) Representation of colocalization changes on TCR-Vβ8.1/mGluR1 after treatment of activated CD8^+^ T cells with CPCCOEt inhibitor. (D) Scatter dot plot displaying the Pearson correlation between TCRVβ8.1/mGluR1 colocalization, and changes after treatment with mGluR1 antagonist. Symbol graphs represent the Mean ± SEM of 7–10 cells ROI per independent experiment (*n* = 3). ^∗^*p* ≤ 0.05, ^∗∗^*p* ≤ 0.01. Two-way ANOVA with multiple comparisons. For a Figure360 author presentation of Figure 4, see https://doi.org/10.1016/j.isci.2025.112772#mmc5.

Our data on the critical role of GluR signaling in T cell activation prompted us to investigate whether TCR and surface mGluR1 colocalize during activation, and if this colocalization is needed to elicit the full activation of T cells. We used confocal microscopy to evaluate the co-occurrence of TCR and mGluR1 on CD8^+^ T cells in both naive and activated cells at 48 h. The TCR Vβ8.1 chain expression and Lck phosphorylation were used as controls for T cell activation (Figure 4A).

We observed increased expression of TCR/Vβ8.1, mGluR1, and phosphorylated Lck (pLck) in activated CD8^+^ T cells compared to naive cells. Treatment with CPCCOEt, the mGluR1 inhibitor, prior to activation prevented an increase in the expression of mGluR1, TCR/Vβ8.1, and pLck (Figure 4B). We also analyzed the localization patterns of these proteins in naive and activated T cells. In naive CD8^+^ T cells, mGluR1 was detected both in the cytoplasm and on the cell membrane, while TCR Vβ8.1 was predominantly on the membrane. However, upon activation, TCR Vβ8.1 and mGluR1 showed increased intensity, with mGluR1 repositioning primarily to the cell surface (Figure 4C). The colocalization analysis revealed that activated CD8^+^ T cells exhibited increased colocalization of TCR Vβ8.1 and mGluR1 on the cell surface. Conversely, pre-treatment of the cells with CPCCOEt prior to activation resulted in the absence of the increased colocalization of TCR Vβ8.1 and mGluR1 in the activated CD8^+^ T cells (Figure 4D). These findings indicate a direct spatial interaction between the TCR and mGluR1 within the same microclusters, establishing an essential role for mGluR1 in the TCR-dependent activation process of CD8^+^ T cells.

### The glutamate receptor engagement impacts the T cell receptor−mediated cascade signaling pathways and rearrangements in energy metabolism

**Figure 5:**
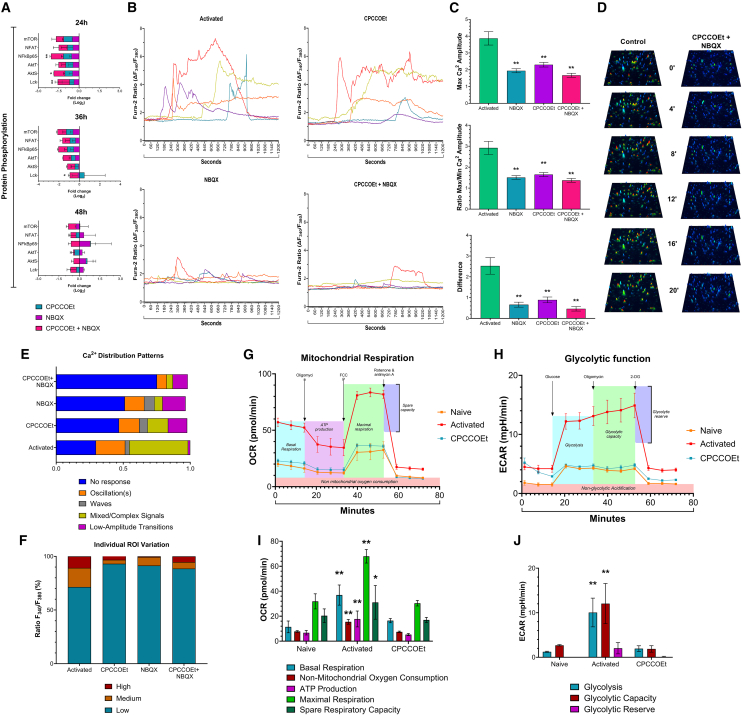
TCR-mediated molecular pathways, metabolism, and calcium firings are impacted by GluR signaling blockade (A) Bar graphs showing changes in protein phosphorylation in CD8⁺ T cells treated with GluR antagonists at 24, 36, and 48 h post-activation. CPCCOEt, non-competitive mGluR1/mGluR5 antagonist; NBQX, competitive AMPA/GluA3 antagonist. Fold changes were calculated relative to untreated activated T cell controls. Horizontal bars represent the Mean ± SEM (*n* = 3). (B) Time-course analysis of calcium ions (Ca^2+^) concentration in activated CD8^+^ T cells treated or untreated with GluR antagonists. The graph is plotted every 5 s for the 20-min duration. Each curve line represents time-dependent Ca^2+^ fluctuations in an individual T cell. (C) The bar graphs depict different parameters of Ca^2+^ changes observed after treatment with GluR antagonists. Bars represent the Mean ± SEM (*n* = 3). (D) Surface dot plots illustrating the dynamic changes of Ca^2+^ fluctuations observed during 20 min after treatment with the combination of the GluR antagonists. (E) Stacked Bar graphs showing the frequency of distinct Ca^2+^ fluctuation patterns in CD8^+^ T cells untreated or treated with GluRs inhibitors. (F) Stacked bar graphs displaying the Ca^2+^ ratio variations in naive and activated CD8^+^ T cells treated or untreated with GluR antagonists at 24 h post-activation. Fura2 (Calcium dye) 340/380 ratios were classified as Low (≤1.5), Moderate (≥1.5 to ≤2), or High (≥2). Data represent 30 cells per 20 min, 5 s cycles. (G) Representative graphs of Seahorse Mitochondrial Stress Test comparing drug-dependent changes in Oxygen Consumption Rate (OCR) of CD8^+^ T cells performed on naive, activated, and activated + mGluR1 inhibitor T cell groups. (H) Representative graphs of Seahorse Glycolysis Test comparing drug-dependent changes in Extracellular Acidification Rate (ECAR) of CD8^+^ T cells performed on naive, activated, and activated + mGluR1 inhibitor groups. (I) Bar graphs showing quantitative changes in different parameters of mitochondrial respiration of CD8^+^ T cells in naive, activated, and activated + mGluR1 inhibitor groups. Bars represent the Mean ± SEM (*n* = 4). (J) Bar graphs showing quantitative changes in different parameters of glycolysis in naive, activated, and activated + mGluR1 inhibitor CD8^+^ T cells. Bars represent the Mean ± SEM (*n* = 4). ^∗^*p* ≤ 0.05, ^∗∗^*p* ≤ 0.01. Unpaired T-test or two-way ANOVA with multiple comparisons. For a Figure360 author presentation of Figure 5, see https://doi.org/10.1016/j.isci.2025.112772#mmc6.

Since the upregulation of CD25, CD69, and CD44 on T cells are linked to productive TCR engagement that triggers downstream signaling pathways leading to full activation,^26^^,^^27^ we determined the role of GluA3 and mGluR1 in supporting TCR-mediated signaling pathways. To determine this, we analyzed the dynamic changes in protein phosphorylation within TCR-mediated signaling pathways at 24 h post-activation in cells treated with GluR antagonists. All analyzed proteins showed reduced expression, with significant decreases in NFκB-p65, Akt, and Lck phosphorylation compared with untreated controls at hours 24 and 36. This effect was most pronounced when mGluR1 and GluA3 were simultaneously blocked (Figure 5A). After 48 h post-activation, the inhibitory effect of individual GluR antagonists on phosphorylation was no longer evident; however, a persistent downregulation was observed in the CD8^+^ T cells treated with both GluRs inhibitors (Figure 5A). Calcium fluxes are another pivotal factor for T cell activation, which generates signals for the nuclear translocation of transcription factors and guides cytoskeleton rearrangement during immune synapse formation.^28^ Therefore, we evaluated if the GluRs blockade affects calcium dynamics in activated CD8^+^ T cells. We evaluated patterns of calcium fluxes in activated and naive CD8^+^ T cells using wide-field microscopy and ratiometric dye Fura-2AM (Figure 5B). Blocking the glutamate receptors in activated CD8^+^ T cells reduced the Max Ca^2+^ amplitude and diminished overall calcium flux patterns under all the treatment conditions (Figures 5C and 5D).

Furthermore, most activated cells treated with the combination of GluR inhibitors showed either absent or markedly reduced calcium oscillations, along with mixed complex signals, suggesting an impaired ability of CD8^+^ T cells to undergo full activation after glutamatergic system blockade (Figure 5E). Furthermore, analysis of individual cell variations revealed a predominance of cells exhibiting low-rank patterns (ratio 340/380 ≤ 1.5) over moderate and high-rank patterns, indicating a systemic reduction in Ca^2+^ signaling across the majority of the CD8^+^ T cells (Figure 5F). These experiments highlight the crucial role of both GluRs in regulating calcium fluxes during CD8^+^ T cell activation. However, to elucidate the functional implications of mGluR1−TCR crosstalk signaling on the metabolic proficiency of CD8^+^ T cells during activation, we employed the Seahorse Metabolic Analyzer to evaluate the impact of mGluR1 inhibition on the transition to glycolysis and the augmentation of mitochondrial respiration, processes essential for the optimal functioning of T cells.

We found that mGluR1 signaling blockade on activated CD8^+^ T cells affects mitochondrial (OXPHOS) and non-mitochondrial (glycolysis) energy production. In contrast, naive cells showed no significant changes in their metabolic energy profiles (Figures 5G and 5H). Notably, the mitochondrial stress test of activated CD8^+^ T cells showed high basal and maximal respiration as well as a robust spare respiratory reserve capacity. (Figure 5I). However, treatment of activated cells with CPCCOEt reduced oxygen consumption rates (OCR) showing similar levels to the naive stage condition, and resulting in diminished ATP production (Figure 5I). Similarly, the glycolysis test showed that only control (untreated) activated CD8^+^ T cells were capable of producing ATP via glycolysis. In contrast, CPCCOEt-treated activated CD8^+^ T cells did not show this capability, displaying metabolic activity comparable to naive condition (Figure 5J).

These results confirm that fully functional mGluR1 signaling is essential for proper CD8⁺ T cell function upon activation. By contrast, the impairment in Ca^2+^ fluxes through GluRs directly disrupts different signaling pathways during the initial stages of TCR-mediated activation, thereby affecting processes such as cell proliferation, differentiation, and acquisition of effector functions that distinguish activated cells from naive T cells.

### Glutamate receptor signaling is necessary for the full effector function of activated CD8^+^ T cells

In order to confirm the role of the glutamatergic system in the acquisition of CD8^+^ T cell functions such as proliferation and cytotoxic capability following activation, we evaluated the effects of blocking GluA3 and mGluR1 with their respective antagonists on these functions.

#### Proliferation

Activated T cells proliferate after TCR engagement, which is critical for an effective cellular immune response.^29^ We observed that mGluR1 is expressed on proliferating CFSE^+^ CD8^+^ T cells, which peaked at 72 h compared to naive controls (Figure S5).

**Figure 6:**
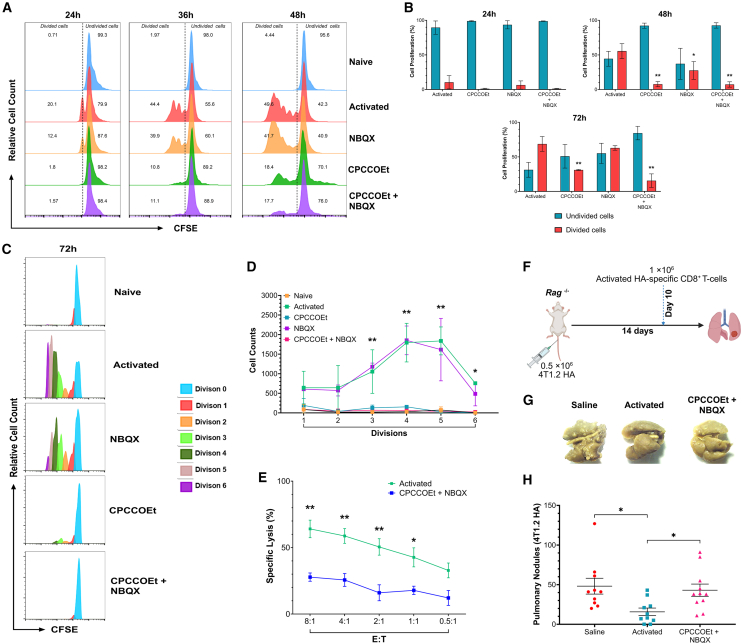
Signaling through glutamate receptors affects CD8^+^ T cell proliferation and cytolytic activity (A) Histograms displaying the effects of GluR inhibitors at 24, 36, and 48 h in activated CD8^+^ T cells; the naive condition was used as a non-proliferative control for this assay. These histogram data are representative of 3 independent experiments. (B) Grouped bar graphs showing the changes in the number of divided/undivided cells post-activation at 24, 36, and 48 h. Bars represent the Mean ± SEM (*n* = 3). (C) Histograms with proliferation modeling algorithm to determine the number of cell divisions after GluR antagonist treatments of activated CD8^+^ T cells for 72 h. These histogram data are representative of 3 independent experiments. (D) Line graph comparison shows the number of cell changes after GluR antagonist treatments. This line graph represents the Mean ± SEM (*n* = 2). (E) Line graph showing the *invitro* CTL assay. The effect of GluR antagonist combination on HA-specific *Rag*2^−/−^Cln4 CD8^+^ T cell effectors against splenocytes labelled with HA or control NP peptide; E:T = effector: target ratio. This line graph represents the Mean ± SEM (*n* = 3). (F) Schematic image depicting the procedure for establishing lung metastatic nodules and T cell transfer in naive Rag2^−/−^ mice. (G) Representative image of the 4T1.2HA lung metastatic nodules on the lung surface from the 3 groups analyzed based on adoptively transferred HA-specific CD8^+^ T cells. (H) Scatter dot graphs displaying the changes in the metastatic nodule counts on tumor-bearing *Rag2*^−/−^ mice that received GluR antagonist-treated HA-specific CD8^+^ T cells in comparison to mice that received activated HA-specific CD8^+^ T; An additional saline group was utilized as a vehicle control. This dot graph represents the Mean ± SEM (*n* = 10 mice per group). ^∗^*p* ≤ 0.05, ^∗∗^*p* ≤ 0.01. Unpaired T-test or two-way ANOVA with multiple comparisons. For a Figure360 author presentation of Figure 6, see https://doi.org/10.1016/j.isci.2025.112772#mmc7.

In contrast, GluA3 expression was not as robust as observed for mGluR1 in the proliferating CFSE^+^ CD8^+^ T cells after 72 h post-activation (Figure S5). The treatment of activated CFSE^+^ CD8^+^ T cells with CPCCOEt, NBQX, or both for 48 h resulted in a significantly reduced proliferation rate (Figure 6A). This anti-proliferative effect was maintained at 72 h post-activation in cells treated with CPCCOEt or combination (Figure 6B). In addition, at 72 h post-activation, treatment with CPCCOEt, or the combination caused a significant reduction in the number of divided cells generated during every cell division (Figures 6C and 6D).

#### Cytotoxicity in vitro

The ability of CD8^+^ T cells to recognize foreign antigens and eliminate targets is critical for infection eradication and control of tumor development. The killing ability of cytolytic CD8^+^ T cells is acquired through a series of events culminating in the production and release of perforin and granzyme B. Furthermore, to evaluate if the lack of signaling through GluR could affect CTL activity, HA-specific CD8^+^ T cells were activated *in vitro* and simultaneously treated with GluR antagonists for 72 h, as previously described. Antigen-specific CD8^+^ T cells treated with a combination of antagonists exhibited a reduced target cell killing compared to activated control cells. Significant differences were observed across the effector-to-target-cell (E:T) ratios; however, the inhibitory effects of GluR antagonist treatment on CD8^+^ T cytolytic function were diminished at lower E:T ratios (Figure 6E).

#### Cytotoxicity in vivo

To evaluate if the effect of GluR signaling on CTLs can be reproduced *in vivo*, we employed metastatic tumor model established in our laboratory. *Rag2*^−/−^ mice received *i.v.* 4TI.2HA breast cancer cells, which form metastatic nodules in the lungs (Figure 6F). After metastatic nodules were established, mice were administered either saline (control) or activated HA-specific CD8^+^ T cells treated with or without GluR double-antagonists, and cytolytic CD8^+^ T cell activity was evaluated by counting metastatic nodules on the lung surface (Figure 6G). As expected, the mice that did not receive the adoptive transfer of antigen-specific CD8^+^ T cells (saline group) exhibited a higher number of metastatic nodules than those that received fully activated CD8^+^ T cells. Furthermore, we observed that the mice receiving CD8^+^ T cells treated with both GluR antagonists had a higher count of metastases than those receiving only activated CD8^+^ T cells (Figure 6H). Collectively, these results show that the GluR blockade impairs the functional ability of CTLs to lyse their targets, underscoring the critical role of GluR signaling in generating a strong effector response against tumors. Moreover, GluR expression emerges as a key determinant of CTL effector function even after their infiltration into tumors.

### mGluR1 expression correlates with CD8^+^ T cell activation and effector memory differentiation in suppressive tumor microenvironment

**Figure 7:**
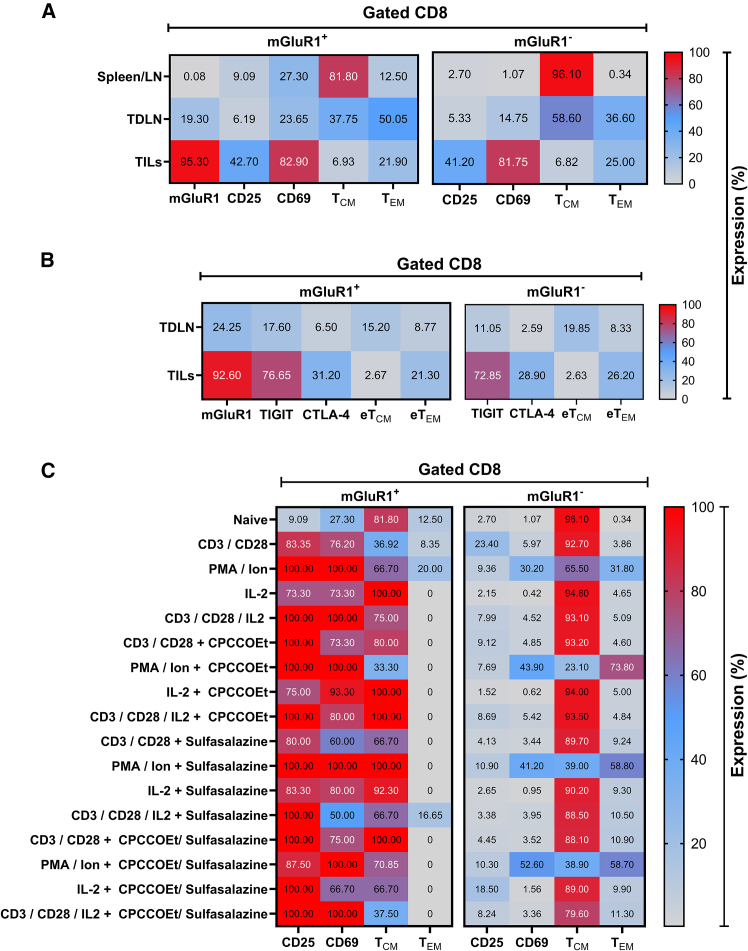
mGluR1 expression is associated with CD8^+^ T cell activation and effector memory differentiation, highlighting its potential to counteract suppressive tumor microenvironment (A) Heatmap showing the percentage expression of activating receptors (CD25, CD69) and the proportions of central memory (T_CM_: CD44^+^CD62L^+^) or effector memory (T_EM_: CD44^+^CD62L^−^) CD8^+^ T cells isolated from spleen, lymph nodes, tumor-draining lymph nodes (TDLN), and tumor-infiltrating lymphocytes (TILs) using CD45^+^ beads, stratified by mGluR1^+/−^ gating. (B) Heatmap shows the percentage expression of inhibitory receptors (TIGIT, CTLA-4) and the proportions of exhausted central memory (eT_CM_: PD1^+^CD62L^+^) and exhausted effector memory (eT_EM_: PD1^+^CD62L^−^) CD8^+^ T cells, also stratified by mGluR1^+/−^ gating. (C) Heatmap displays the expression of activating receptors (CD25, CD69) and the proportions of T_CM_ (CD44^+^CD62L^+^) and T_EM_ (CD44^+^CD62L^−^) purified CD8^+^ T cells from spleen/lymph nodes treated or untreated with the mGluR1 inhibitor (CPCCOEt) and/or xCT inhibitor (Sulfasalazine), using different treatment combinations. All heatmaps are presented as Mean ± SEM from cells from 4 mice (*n* = 4 mice per group). For a Figure360 author presentation of Figure 7, see https://doi.org/10.1016/j.isci.2025.112772#mmc8.

We analyzed the expression of activating receptors on CD8^+^ T cells derived from tumor-infiltrating lymphocytes (TILs) and tumor-draining lymph nodes (TDLNs) (Figure S6) ten days post-tumor inoculation. CD8^+^ T cells exhibited the elevated expression of mGluR1 in both TDLNs and TILs compared to baseline levels in the spleen and peripheral lymph nodes. Notably, the activating receptors CD25 and CD69 were upregulated at the tumor site, accompanied by an increased population of effector memory T cells (T_EM_) (Figure 7A). Inhibitory receptors (Figure S7), TIGIT and CTLA-4, were significantly upregulated in tumor-infiltrating lymphocytes compared to those in TDLNs. Furthermore, PD-1 expression analysis, in conjunction with CD62L gating, revealed that T_EM_ cells maintained higher PD-1 expression relative to central memory T cells (T_CM_) (Figure 7B). These findings highlight key dynamics in mGluR1^+^CD8^+^ T cell behavior during antitumor responses.

Next, we conducted expression analysis of the CD8^+^ T cell activation receptors, using alternative activation strategies (PMA/Ionomycin or IL-2) and combinations of inhibitors targeting mGluR1 (CPCCOEt) and xCT (Sulfasalazine). CD8^+^ T cells expressing mGluR1 exhibited elevated levels of CD25 and CD69 receptors compared to the total CD8^+^ T cell population (Figure 7C). We employed a linear regression analysis of expression data to forecast the role of mGluR1 expression in the activation of CD8^+^ T cells (Table 1). We found that an increase in CD25 and CD69 expressions is tightly linked with mGluR1 expression irrespective of the activation strategy employed. However, other parameters, such as an increase in the effector memory CD8^+^ T cell population, showed a stronger correlation with PMA/Ionomycin-mediated activation (*p = 0.0019*) and the presence of mGluR1 (*p = 0.0026*) compared to other activation strategies. These findings suggest that mGluR1 plays a pivotal role in enhancing the expression of specific activation receptors, independent of the method used for the activation of CD8^+^ T lymphocytes.

**Table 1:** Activation method-dependent CD8^+^ T cell activating marker regression model

Variable (Y)	Variable (X)	Multiple R	R^2^ value	Adjusted R^2^ value	F (DFn, DFd)	*p* value	Predictive Outcome
CD25	mGluR1 CD3/CD28 PMA/Ionomycin IL-2	0.9510	0.9044	0.8912	F (1, 29) = 261.3 F (1, 29) = 9.948 F (1, 29) = 10.33 F (1, 29) = 4.694	<*0.0001* *0.0037* *0.0032* *0.0386*	**↑↑** Expression
CD69	mGluR1 CD3/CD28 PMA/Ionomycin IL-2	0.9555	0.9131	0.9011	F (1, 29) = 255.2 F (1, 29) = 3.300 F (1, 29) = 40.18 F (1, 29) = 4.693	<*0.0001* *0.0796* <*0.0001* *0.0386*	**↑↑** Expression
T_CM_	mGluR1 CD3/CD28 PMA/Ionomycin IL-2	0.5978	0.3574	0.2688	F (1, 29) = 0.4302 F (1, 29) = 1.659 F (1, 29) = 10.25 F (1, 29) = 0.0061	0.5171 0.2079 *0.0033* 0.9378	**↑** Expression
T_EM_	mGluR1 CD3/CD28 PMA/Ionomycin IL-2	0.7325	0.5365	0.4726	F (1, 29) = 10.86 F (1, 29) = 0.0472 F (1, 29) = 11.70 F (1, 29) = 0.0018	*0.0026* 0.8294 *0.0019* 0.9656	**↑** Expression

Table displaying the predictive estimates and the positive influence of the independent variable X [mGluR1; CD3/CD28; PMA/Ionomycin; IL-2] on the dependent variable Y [CD25; CD69; T_CM_; T_EM_]. The significance-associated *p* values are italicized. Additionally, the directional arrows illustrate the predictive outcomes of the positive influence of variable X on variable Y after LL/2 tumor exposure. Dependent variable: Y; Independent variables: X (mGluR1 [β1]; CD3/CD28[β2]; PMA/Ionomycin [β3]; IL-2 [β4]); Regression Model Formula: Y = β0+ β1+ β2 + β3 + β4 (X).

These findings provide insights into the potential mechanisms underlying the enhanced antitumor efficacy of CD8^+^ T cells and highlight the pivotal role mGluR1 plays in regulating antitumor activities of CD8^+^ T cells. Data also emphasize the tumor microenvironment as a restricting factor suppressing the activities of the tumor-infiltrating and tumor-draining lymph node (TDLN) CD8^+^ T-cells that can be countered by the mGluR1 expression levels on T cells.

## Discussion

Glutamate is a non-essential amino acid that acts as both a substrate and a product in many reactions within mammalian cells^30^ and is involved in multiple metabolic pathways synthesizing amino acids, nucleic acids, nucleotides, and metabolites.^1^^,^^18^^,^^31^ Notably, glutamate serves as the brain’s principal excitatory neurotransmitter and plays a pivotal role in cognitive functions within the central nervous system (CNS).^10^ However, excessive signaling through glutamate receptors can lead to excitotoxicity, which is linked to the development of neurodegenerative disorders.^32^ Although neurotransmitter receptors are typically associated with the CNS, they have also been identified in other organ systems,^33^ suggesting that the nervous and other body systems can communicate and impact each other’s functions.^11^^,^^33^ Over the past decade, glutamate has also been characterized as an immunomodulator and a critical regulator of tumor growth.^34^

Recent research has indicated that glutamate production is not confined to the brain but occurs in other tissues containing glutamatergic systems, including immune cells.^10^^,^^35^ Lymphocytes, recognized as a pivotal immune response component, express GluRs, glutamate transporters, and are capable of secreting glutamate.^11^

Our results indicate that subsets of CD4^+^ and CD8^+^ T cells from the spleen and lymph nodes express certain GluRs, primarily GluA3 and mGluR1, whereas others, such as mGluR5, are expressed at low levels. This finding is intriguing, as previous studies have shown that in other immunological sites, such as the thymus, thymocytes—developing T cells—express significant levels of GluRs, including mGluR5.^36^^,^^37^ These studies also suggest that such receptors may mediate maturation during positive and negative selection. Based on this, we propose that once T lymphocytes mature and leave the thymus, the expression of mGluR5 decreases. This aligns with studies evaluating GluR expression, including mGluR5, in human PBMCs, which demonstrated that while mGluR5 maintains constitutive expression at the mRNA level, its protein levels remain low.^38^^,^^39^ In addition, CD11C^+^ APCs identified in both the spleen and lymph nodes demonstrated minimal expression of all glutamate receptors (GluRs). This finding is consistent with previous research indicating the low expression of mGluR1 and mGluR5 in monocyte-derived APCs.^39^ Conversely, thymic APCs originating from the medullary region exhibited elevated levels of GluRs, including mGluR5.^37^

These observations suggest that GluR expression varies among APCs from different tissues and may be linked to sustained antigen presentation in specific thymic regions during negative selection. By contrast, in peripheral tissues, such as the spleen or blood, where self-antigen presentation is limited, the expression of these receptors remains minimal.

We also observed that the expression of GluRs is tightly linked to T cell activation in both *in vivo* (murine breast cancer model) and *in* *vitro* (TCR stimulation) systems. Our investigation involving mice bearing breast tumors uncovered that TIL generally diminished the expression of specific GluRs, particularly mGluR1. However, upon stratifying between activated and non-activated infiltrating cells based on the expression of CD25 or CD44 activation markers, we observed heightened levels of GluRs on activated TIL in comparison to the naive CD4^+^ and CD8^+^ T cells from control mice. Interestingly, there is evidence of increased glutamate concentrations in pathological conditions, particularly cancer. Notably, plasma glutamate levels are increased in the blood of patients with lung, or head and neck cancers compared to healthy individuals.^40^ Furthermore, heightened glutamate concentration seems to increase with the severity of different tumor types and non-neurological diseases,^3^^,^^22^ suggesting a dysregulation in the glutamate processing mechanisms essential for cells through GluRs. Our results indicate that within the tumor microenvironment, although GluR expression is linked to T cell activation, additional factors may downregulate certain GluR expression, thereby limiting glutamate processing capacity and impairing antitumor responses.

The levels of metabotropic glutamate receptors mGluR1 and mGluR5 are higher in activated human T cells when compared to naive T cells, suggesting a potential involvement of GluR signaling in the activation and proliferation of CD8^+^ T cells.^38^ It is noteworthy that human T cells, B cells, and APCs can also express AMPA receptors.^3^^,^^21^ Our *in vitro* findings corroborate the increased expression of GluRs in activated CD25^+^CD44^+^ CD4^+^ and CD8^+^ T cells relative to their naive counterparts. This augmented expression is not specific to mGluRs, AMPA, or NMDA receptors but reflects a general trend reported by other studies.^10^^,^^22^ Notably, surface GluR expression in murine T cells is contingent upon the activation state of T cells and reaches its peak approximately 48 h post-TCR activation. Moreover, our observations in CD8^+^ T cells revealed that early activation receptors, such as CD69, exhibit sustained higher expression in GluR-expressing cells, supporting an active involvement of GluR signaling during the early phases of CD8^+^ T cell activation.

Glutamine from the extracellular space is essential for activating T cells, serving as their primary energy source during proliferation and cytokine production. Once internalized into cells, glutamine is converted to glutamate.^18^ Previous studies have reported that during T cell activation, APCs (including DCs and macrophages) are the main source of glutamate, which binds to mGluR1, supporting the T cell to continue its activation process in the lymph nodes.^38^^,^^39^ In contrast, our study demonstrated that in the absence of glutamate-derived APCs, CD4^+^ and CD8^+^ T cells tend to increase glutamate secretion at 24 h and express molecules from the glutamatergic system with significant changes at 72 h post-activation. Our findings align with a study showing that antigen-stimulated T cells secrete glutamate, which can subsequently be imported by other cells within the coculture.^41^

The glutamate transporter, antiporter, and glutaminase expression has also been identified in both human and mouse immune cells. Their function has been linked to extracellular glutamate secretion and intracellular synthesis of glutathione, an essential antioxidant during T cell proliferation.^12^^,^^22^^,^^42^^,^^43^ Since T cells can secrete glutamate into the extracellular space, we investigated if these cells express glutamate transporter, antiporter, and glutaminase, the enzyme that converts glutamine into glutamate. We found that activated CD8^+^ T cells constitutively expressed the enzyme glutaminase and the neutral amino acid transporter SLC1A5 (ASCT2), confirming that these proteins are needed for the naive T cell to achieve full activation.^18^^,^^19^^,^^44^^,^^45^ While other studies have reported that these proteins are increased only after T cell activation,^46^^,^^47^ we found that glutaminase and SLC1A5 are present at the same levels in both naive and activated CD8^+^ T cells. At the same time, activated CD8^+^ T cells exhibited higher expression of cystine/glutamate antiporter (xCT) and glutamate transporter 1 (GLT1), consistent with enhanced intracellular glutamate production. Moreover, the expression of these glutamate antiporters and transporters increased upon T cell activation. While little is known about the presence of GLT1 in T cells,^48^ xCT has been described as crucial for T cell proliferation due to the high demand of cystine/cysteine in glutathione and protein synthesis.^49^^,^^50^

To investigate the relationship between TCR and GluR, we employed two GluR antagonists: NBQX, a competitive AMPA antagonist that blocks glutamate binding site, and CPCCOEt, a non-competitive Group 1 metabotropic antagonist, which inhibits receptor signaling without affecting glutamate binding.^25^^,^^51^ These antagonists impede signaling through GluA3 and mGluR1 (together with mGluR5) but do not interfere with the TCR-mediated activation or glutamine metabolism.^18^ We showed that the antagonist treatment of CD8^+^ T cells resulted in delayed activation without affecting their cell viability. This delayed cell activation and consequent expression of surface activation markers^52^^,^^53^^,^^54^ were affected by a single GluR antagonist treatment up to 36 h post-activation. In contrast, after blocking both AMPA and group 1 mGluRs, the decreased expression of CD69, and CD25 markers persisted up to 48 h.

Since glutamate is part of the energetics pathway required for T cell activation, we investigated if blocking access to glutamate would produce the same effect as blocking the receptor signaling with the GluR antagonists. Interestingly, similar to the reduced expression of activation markers after the inhibition of GluR signaling, CD8^+^ T cells exhibited diminished activation due to the glutamate arrest. However, subsequent cell death was observed after the absence of glutamate, which can be attributed to the crucial role of glutamate as an essential amino acid for metabolism and cell differentiation.^19^^,^^55^ Therefore, our experimental findings, which involved blocking glutamate receptors without impacting cell viability, provide conclusive evidence of the pivotal role played by GluR-mediated signaling beyond energy regulation, suggesting a direct involvement of GluR-mediated signaling in regulating TCR function and its associated signaling pathways.

The activation of T cells is a highly coordinated process that involves multiple pathways, including calcium signaling, nuclear translocation of NF-κB, NFAT, and activation of the PI3K–AKT–mTOR axis.^56^ During these processes, TCR engagement by peptide-MHC ligands induces a cascade of signaling events culminating in the proliferation and acquisition of effector functions.^15^^,^^57^ In our study, blocking GluR signaling reduced the phosphorylation of key proteins involved in T cell activation pathways (Lck, Akt, NFκBp65, NFAT, and mTOR) as early as 24 h post-activation; however, this effect was transient and completely abrogated at 48 h. Decreased phosphorylation of these proteins in TCR-activated and GluR antagonist-treated CD8^+^ T cells confirmed diminished cell activation and the importance of the GluR signaling as another stimulatory pathway.

We further confirmed this hypothesis by comparing intracellular calcium (Ca^2+^) firing in naive and activated cells. Activated CD8^+^ T cells treated with CPCCOEt antagonist displayed patterns of variations similar to naive untreated CD8^+^ T cells. In parallel, metabolic reprogramming is initiated during the activation of T cells,^58^ where glucose uptake and utilization increase to meet the high-energy demands and is usually achieved through mechanisms such as anaerobic glycolysis, OXPHOS, increased growth factor cytokines such as interleukin-2 (IL-2) signaling or the activation of AKT by the ligation of costimulatory CD28 receptor.^59^^,^^60^ Our data also indicated that blocking GluRs decreases energy acquisition, revealing a critical previously unreported role of the mGluR1 receptor in the glycolytic function of activated CD8^+^ T lymphocytes.

The disruption of these signaling pathways and metabolism following GluR blockade with antagonists has pointed to the existence of a crosstalk between the TCR complex and GluRs. This was confirmed through confocal microscopy image analysis, unveiling the spatial colocalization of mGluR1 and TCR Vβ8.1, with markedly higher prevalence in activated CD8^+^ T cells compared to naive cells and activated CD8^+^ T cells impacted by mGluR1 antagonist treatment.

The coordinated interplay among these pathways is indispensable for various effector functions in T cells, encompassing metabolism, proliferation, differentiation, cytokine secretion, Ca^2+^ signaling and cytotoxicity.^16^^,^^28^^,^^61^ Under normal conditions, an activated CD8^+^ T cell exhibits substantial proliferative capacity, consistently producing cytokines that support its effector function following TCR-dependent activation.^15^ Based on our data on the spatial interaction between TCR and mGluR1 and the signaling pathways altered after GluR blocking in CD8^+^ T cells, we evaluated the involvement of GluR signaling in T cell effector functions.

Previous studies have suggested a correlation between GluR signaling, T cell activation, and proliferation.^39^ Our experiments revealed that blocking GluA3 and mGluR1 signaling in activated cells resulted in a drastic decrease in proliferation. Furthermore, our findings indicate that CD8^+^ T cells’ ability to lyse target cells through perforin-granzyme B^62^ was also reduced *in vitro* and *in vivo* after the blockade of GluR signaling with double antagonist treatment. The diminished capacity of CTL effectors to eradicate lung metastasis following GluR signaling blockade underscores the significant impact of the TCR-GluR signaling pathway on the antitumor immune response.

Recent research has been delving into the metabolic pathways that shape tumor progression and T cell immune responses.^63^ In this work, we provide compelling evidence for the role of GluR signaling during TCR-dependent activation. Specifically, mGluR1 has been identified as capable of directly interacting with T cell receptors and influencing molecular signaling pathways and metabolism, thereby regulating CTL proliferation and cytotoxicity. In addition, our mathematical analysis highlighted key CD8^+^ T cell behavior dynamics during antitumor responses. As CD8^+^ T cells migrate toward the tumor microenvironment (TME), their activation potential increases, as indicated by heightened CD69 expression, potentially supported by mGluR1 expression and an enhanced generation of T_EM_ populations in TDLNs. However, these activated lymphocytes also demonstrate high sustained expression of inhibitory receptors such as TIGIT and CTLA-4, which play critical roles in maintaining immune homeostasis under physiological conditions and are associated with early tumor stages in other cancer types.^64^ Interestingly, T_EM_ cells infiltrating the tumor exhibited persistently elevated PD-1 expression compared to their counterparts in TDLNs. This is significant as effector memory T cells are proposed to possess potent cytotoxic activity yet remain vulnerable to inhibitory signaling within an immunosuppressive TME scenario.^65^ Consequently, mGluR1 expression in CD8^+^ T cells may facilitate their activation and functional differentiation. However, additional variables such as the expression of inhibitory ligands such as PD-L1 or CD155 within the TME may impair CD8^+^ T cell cytotoxic activity, particularly in tumors exhibiting resistance to immune-mediated elimination. Nevertheless, a comprehensive analysis utilizing a conditional model of mGluR1 in T cells is imperative and remains a consideration for future research within this field.

These insights underscore the significance of understanding the interplay between the nervous and immune systems, exemplified by the interaction between the neurotransmitter glutamate and CD8^+^ T cells. This perspective enriches our comprehension of T cell immune responses, particularly in conditions such as cancer, where the immune system is compromised. Furthermore, it reveals broader opportunities for the development of potential neuroimmunological therapeutics across other conditions, including autoimmune diseases, neuroimmunopathologies, and infectious diseases.

Our study highlights the fundamental role of the mGluR1 receptor as a critical contributor to TCR-dependent activation, which presents significant glutamatergic therapeutic potential across oncology, autoimmune diseases, neuroimmunopathologies, and infectious diseases. Numerous studies emphasized the significance of the glutamatergic system in certain tumors.^20^^,^^63^^,^^66^ Based on these studies, more refined therapeutic strategies may be developed to enhance the activation of CD8^+^ T cells through the modulation of mGluR1. This could lead to improved antitumor responses, particularly when combined with cell therapy approaches. Additionally, integrating mGluR1 modulation with existing immunotherapies, such as immune checkpoint inhibitors, may increase their efficacy, especially in immune-resistant tumor microenvironments.

For autoimmune diseases, the involvement of mGluR1 in T cell activation suggests that the selective inhibition of this pathway may facilitate the modulation of hyperactive immune responses.^67^ This approach could reduce inflammation while preserving essential immune functions. In neuroimmunology, the interaction between glutamate axis signaling and immune responses suggests potential applications for treating neuroinflammatory disorders.^68^^,^^69^^,^^70^ Targeting mGluR1 in immune cells may offer effective therapeutic strategies for treating conditions such as multiple sclerosis and Alzheimer’s disease, which involve both immune and neuronal components. Additionally, in infectious diseases, enhancing T cell function through mGluR1 activation could fortify immune responses against chronic infections that evade immunity, including intracellular viral and bacterial infections.^71^

In summary, exploring alternative mechanisms to modulate antitumor immunity *via* central nervous system messengers is a burgeoning area of research. This approach has exhibited promise in various tumor models, including melanoma and lung cancer.^72^^,^^73^ The complex interplay between the nervous and immune systems holds considerable potential for the development of more targeted and effective therapies for cancer and other immunosuppressive conditions.

### Limitations of the study

This investigation offers compelling evidence for the intrinsic role of glutamate receptors in cytotoxic T cells, highlighting their significance as key co-stimulators in TCR-mediated activation. This contributes to enhanced effector responses against immunological challenges such as cancer. Although these findings demonstrate that the disruption of GluRs diminishes the functional capacity of CD8^+^ T cells, certain aspects remain unresolved, such as the impact of the tumor microenvironment on GluR expression, warranting further exploration in future research using T cell GluR conditional knockout mice. In addition, these applications need further research to refine the therapeutic strategies aimed at targeting mGluR1. This could involve the development of specific agonists, antagonists, or modulators, followed by the rigorous testing of their efficacy and safety in relevant preclinical and clinical settings.

## Resource availability

### Lead contact

For all inquiries regarding resources and reagents, please contact the correspondence contact, Anil Shanker (ashanker@mmc.edu).

### Materials availability

This study did not produce any new, unique reagents.

### Data and code availability

• All data reported in this article will be shared by the lead contact upon request.
• This article does not utilize any assessment code to conduct this research.
• Any additional information required to reanalyze the data reported in this article is available from the lead contact upon request.

## Acknowledgments

The article presented here comprises part of the studies presented by TWH in his thesis under the supervision of AS for the award of the degree of Master of Science at Meharry Medical College. We thank T. Rana, J. Tonello, M. Aksu and O. Korolkova for their invaluable input on the article. The funding for this work was received by AS through several 10.13039/100000002National Institutes of Health (NIH) grants, specifically U54 CA163069, U54 MD007593, SCI CA182843, and U54 MD007586. Furthermore, this research was conducted at the Meharry Medical College Core Facilities, which receive support from 10.13039/100000002NIH grants MD007586, CA163069, and S10RR025497.

## Author contributions

Conceptualization, A.S., and T.W.H.; data curation, T.W.H., M.T.P.d.A., R.V.U., S.G.O., J.S.G., T.K., M.A.M., and A.V.I.; formal analysis, T.W.H., A.S., M.T.P.d.A., R.V.U., S.G.O., J.S.G., T.K., and A.V.I.; funding acquisition, A.S.; investigation, A.S., T.W.H., M.T.P.d.A., R.V.U., S.G.O., and T.K.; methodology, A.S., T.W.H., M.T.P.d.A., R.V.U., S.G.O., J.S.G., T.K., and A.V.I.; resources, A.S.; supervision, A.S.; validation, M.T.P.d.A., R.V.U., S.G.O., J.S.G., T.K.; writing – original draft, T.W.H., A.S., M.T.P.d.A.; writing – review and editing, A.S., T.W.H., M.T.P.d.A., R.V.U., S.G.O., J.S.G., T.K., and A.V.I.

## Declaration of interests

The authors declare no competing interests.

## STAR★Methods

### Key resources table

**Table:** 

REAGENT or RESOURCE	SOURCE	IDENTIFIER
**Antibodies**		

TruStain FcX™	Biolegend®	(Cat# 101320, RRID: AB_1574975)
Anti-Glutamate Receptor 3 Antibody, clone 3B3	Sigma-Aldrich	(Cat# MAB5416, RRID: AB_2113897)
Anti-mGluR1	Novus Biologicals R&D systems	(Cat# NBP1-50203, RRID: AB_10012403)
Anti-mGluR5	Novus Biologicals R&D systems	(Cat# MAB4514, RRID: AB_2232846)
Anti-NR1	Novus Biologicals R&D systems	(Cat# PPS083, RRID: AB_2112007)
Anti-NR2B [pTyr1336]	Novus Biologicals R&D systems	(Cat# PPS057, RRID: AB_2112917)
FITC Donkey anti-rabbit IgG	Biolegend®	(Cat# 406403, RRID: AB_893531)
APC anti-mouse IgG1 Antibody	Biolegend®	(Cat# 406610, RRID: AB_10696420)
FITC anti-mouse IgG1 Antibody	Biolegend®	(Cat# 406606, RRID: AB_493293)
APC anti-mouse CD3 Antibody	Biolegend®	(Cat# 100236, RRID: AB_2561456)
PerCP/Cyanine5.5 anti-mouse/human CD11b Antibody	Biolegend®	(Cat# 101228, RRID: AB_893232)
PerCP/Cyanine5.5 anti-mouse CD4 Antibody	Biolegend®	(Cat# 100434, RRID: AB_893324)
PE anti-mouse CD4 Antibody	Biolegend®	(Cat# 100408, RRID: AB_312693)
APC anti-mouse CD4 Antibody	Biolegend®	(Cat# 100412, RRID: AB_312697)
FITC anti-mouse CD4 Antibody	Biolegend®	(Cat# 100406, RRID: AB_312691)
PerCP/Cyanine5.5 anti-mouse CD8 Antibody	Biolegend®	(Cat# 100734, RRID: AB_2075238)
PE anti-mouse CD8a Antibody	Biolegend®	(Cat# 100708, RRID: AB_312747)
APC anti-mouse CD8a Antibody	Biolegend®	(Cat# 100712, RRID: AB_312751)
FITC anti-mouse CD8a Antibody	Biolegend®	(Cat# 100706, RRID: AB_312745)
APC anti-mouse CD44 Antibody	Biolegend®	(Cat# 103012, RRID: AB_312963)
FITC anti-mouse CD44 Antibody	Biolegend®	(Cat# 103006, RRID: AB_312957)
FITC anti-mouse CD25 Antibody	Biolegend®	(Cat# 102006, RRID: AB_312855)
PE anti-mouse CD25 Antibody	Biolegend®	(Cat# 102008, RRID: AB_312857)
APC anti-mouse CD25 Antibody	Biolegend®	(Cat# 101910, RRID: AB_2280288)
PE/Cyanine7 anti-mouse CD62L Antibody	Biolegend®	(Cat# 104418, RRID:AB_313103)
APC anti-mouse TIGIT (Vstm3) Antibody	Biolegend®	(Cat# 156106, RRID:AB_2750515)
CTLA-4 Monoclonal Antibody (1B8), FITC	Thermo fisher	(Cat# HMCD15201, RRID:AB_2536593)
PerCP/Cyanine5.5 anti-mouse CD69 Antibody	Biolegend®	(Cat# 104522, RRID: AB_2260065)
Anti-Lck, phospho (Tyr505) Antibody, Unconjugated	Cell signaling	(Cat# 2751, RRID: AB_330446)
Phospho-Akt (Thr308) (C31E5E) Rabbit mAb	Cell signaling	(Cat# 2965, RRID: AB_2255933)
Unconjugated anti-rabbit AKT	Cell signaling	(Cat# 4685, RRID: AB_2225340)
Unconjugated anti-rabbit NFκBp65	Cell signaling	(Cat# 8242, RRID: AB_10859369)
Unconjugated NFAT1 (phospho Ser54)	GeneTex	(Cat# GTX25246, RRID: AB_380487)
Unconjugated SLC1A5 Antibody	Novus Biologicals R&D systems	(Cat# NBP1-89327, RRID: AB_11024237)
Anti-L Glutamate antibody	Abcam	(Cat# ab9440, RRID: AB_307256)
Rabbit IgG Isotype Control	Novus Biologicals R&D systems	(Cat# NB810-56910, RRID: AB_844243)
Purified anti-mouse TCR Vβ8.1, 8.2	Biolegend®	(Cat# 118402, RRID: AB_1027707)
Goat anti-Mouse IgG (H+L) Secondary Antibody, DyLight™ 405	Thermo fisher	(Cat# 35501BID, RRID: AB_2533209)
Goat anti-Rabbit IgG (H+L) Cross-Adsorbed Secondary Antibody, Alexa Fluor™ 555	Thermo fisher	(Cat# A-21428, RRID: AB_2535849)
mGluR1 Antibody [FITC]	Novus Biologicals R&D systems	(Cat# NB100-93555F, RRID: AB_3174610)
xCT Antibody [PE]	Novus Biologicals R&D systems	(Cat# NB300-318PE, RRID: AB_3187394)
SLC1A2 / EAAT2 / GLT-1 Antibody [APC]	LS Bio	(Cat# LS-C516967-100, RRID: AB_3361348)
GLS Antibody [PE]	LS Bio	(Cat# LS-C728142-100, RRID: AB_3361703)
Phospho-LCK (Tyr505) Monoclonal Antibody (SRRCHA)	eBioscience™	(Cat# 50-9076-42, RRID: AB_2574315)
AlexaFluor 555 goat anti-rat IgG	Invitrogen	(Cat# A-21434, RRID: AB_2535855)
Goat Anti-Mo IgG DyLight 405	Invitrogen	(Cat# 35500BID, RRID: AB_2533208)

**Biological samples**		

Mouse Spleen	This paper	N/A
Mouse Lymph Nodes	This paper	N/A

**Chemicals, peptides, and recombinant proteins**		

MACS® Tissue Storage Solution	Miltenyi Biotec	Cat# 130-100-008
RPMI 1640 Medium	Gibco™	Cat# 11875093
Fetal Bovine Serum, certified, United States	Gibco™	Cat# 16000044
Isoflurane	Sigma-Aldrich	Cat# 792632
ACK Lysing Buffer	Thermo fisher	Cat# 50-101-8893
CD4^+^ T-Cell Isolation Kit, mouse	Miltenyi Biotec	Cat# 130-104-453
CD8a+ T-Cell Isolation Kit, mouse	Miltenyi Biotec	Cat# 130-104-075
CD45 (TIL) MicroBeads mouse	Miltenyi Biotec	Cat# 130-110-618
Dynabeads™ Mouse T-Activator CD3/CD28 for T-Cell Expansion and Activation	Thermo fisher	Cat# 11456D
NBQX	Tocris Bioscience	Cat# 0373
CPCCOEt	Tocris Bioscience	Cat# 1028
Sulfasalazine	Thermo fisher	Cat# 461240050
Glutamate Assay Kit	Abcam	Cat# ab138883
BD Cytofix/Cytoperm™ Fixation/Permeabilization Kit	BD Biosciences	Cat# 554714
7-AAD (7-Aminoactinomycin D)	Biolegend	Cat# 420404
Cell Trace™ CFSE Cell Proliferation Kit, for flow cytometry	Thermo fisher	Cat# C34554
Fura-2-acetoxymethyl ester (Fura-2, AM), cell permeant	Thermo fisher	Cat# F1221
Perm/Wash Buffer	BD Biosciences	Cat# 554723
PBS, pH 7.4	Gibco™	Cat# 10010049
Triton™ X-100	Millipore-Sigma	Cat# 9036-19-5
ProLong™ Diamond Antifade Mountant	Thermo fisher	Cat# P36965
Poly-D-Lysine	Thermo fisher	Cat# A3890401
Collagenase type 2	Worthington Biochemical	Cat# LS004174
SRB Assay / Sulforhodamine B Assay Kit	Abcam	Cat# ab235935
IL-2/Fc chimera mouse recombinant	SIGMA	Cat# I 9905
Phorbol 12-myristate 13-acetate	Milipore Sigma	Cat# 79346
Ionomycin calcium salt	Milipore Sigma	Cat# I0634

**Critical commercial assays**		

Seahorse XF Cell Mito Stress Test Kit	Agilent Technologies	Cat# 103015-100
Seahorse XF Glycolysis Stress Test Kit	Agilent Technologies	Cat# 103017-100

**Experimental models: Cell lines**		

4T1.2HA	Suzanne Ostrand-Rosenberg (University of Maryland)	N/A

**Experimental models: Organisms/strains**		

Mouse: Balb/c (Wild type)	Name/Meharry Medical College (MMC)	N/A
Mouse: Balb/c *Rag2*^-/-^	Name/Meharry Medical College (MMC)	N/A
Mouse: Balb/c *Rag2*^-/-^ /Cln4^+^	Name/Meharry Medical College (MMC)	N/A

**Software and algorithms**		

FlowJo v10.9.	FlowJo, LLC	https://www.flowjo.com/solutions/flowjo/downloads
GraphPad Prism v9.5.1	GraphPad Software, LLC	https://www.graphpad.com/
NIS-Elements Imaging Software	Nikon	5.21.0.14830
Seahorse Wave Controller Software	Agilent Technologies	2.6.3

**Other**		

Countess Automated Cell Counter	Invitrogen	I-CACC
Cell Strainers	Corning®	Cat# 431750
Synergy H1 Plate Reader	BioTek	N/A
Guava® easyCyte	Millipore	0500-4020
Poly-L-Lysine coated adhesive microscope slides	Sigma	Cat# P6407
Nikon Eclipse TE 2000E wide-field microscope	Nikon	N/A
Electron Microscopy Sciences Poly-L-Lysine Coated Slides, 72/bx	Fisher Scientific	Cat# 50-279-88
Eclipse TE2000 Inverted Microscope	Nikon	N/A
A1 HD25 Confocal Microscope System	Nikon	N/A
Extracellular Flux Analyzer XF96 Seahorse	Agilent Technologies	N/A

### Experimental model and study participant details

#### Mouse models

Mice 6-8 weeks old were used in the study unless indicated otherwise. For experiments using 4T1 or 4T1.2HA orthotopic model, Balb/c WT female mice were used unless indicated otherwise Balb/c WT mice were purchased from Harlan Laboratories (Indianapolis, IN). Balb/c, Balb/c Rag2^-/-^ and Balb/c Rag2^-/-^/Cln4 mice (αβ TCR specific for hemagglutinin HA518–526 peptide restricted to H-2K^*d*^) bred locally at Meharry Medical College (MMC) animal facility and housed under pathogen-free conditions. Mice were cared for in accordance with the procedures outlined in the National Institutes of Health and Institutional Animal Care and Use Committee (IACUC) protocol at MMC (Animal protocol #16-07-582). This institution is accredited by the Association for Assessment and Accreditation of Laboratory Animal Care International and follows the Public Health Service Policy for treating and using laboratory animals under pathogen-free conditions.

#### Mouse tumor cell lines

The murine mammary adenocarcinoma cell line 4T1.2HA (courtesy Suzanne Ostrand-Rosenberg, University of Maryland, Baltimore, MD) was maintained in 10% FCS-supplemented standard RPMI-1640 (Gibco, Invitrogen) medium. Low-passage (< 5) tumor cell cultures were used for the experiments.

### Method details

#### Orthotopic tumor establishment

Solid tumors were established in syngeneic Balb/c WT by injecting 2 × 10^6^ 4T1.2HA cells orthotopically into the mammary fat pads. Following establishing palpable tumors of approximately 120 mm^3^, mice were euthanized, tumors excised, and TILs isolated. To induce lung metastasis, 0.5× 10^6^ 4T1.2HA were *i.v.* injected into Rag2^-/-^ mice. 14 days post-injection, mice were euthanized, and the lungs were harvested for metastasis assessment.

#### Cell harvesting, preparation, and count

For tissue harvesting, mice were anesthetized with isoflurane (Millipore Sigma, St Louis, MO), followed by cervical dislocation. Single-cell suspensions were prepared by gently pressing spleen and lymph nodes in RPMI media through 40μm cell strainers (Corning, Corning, NY), followed by transferring to 15 mL conical tubes and spinning down at 2000 rpm for 5 min at 4°C. Cells were washed twice, resuspended in 1 ml of ACK erythrocytes lysis buffer (KD Medical, Columbia, MD), incubated for 1 min at room temperature, and centrifuged in a large volume of complete RPMI media. The pellet was once again resuspended in complete RPMI media. An automated cell counter (Invitrogen-Thermo Fisher Scientific, Waltham, MA) was used to measure total cell count and viability.

#### Lung metastasis count

Adult Balb/c Rag2^-/-^ mice (8-12-week-old) were divided into three groups, received intravenously 0.5 × 10^6^ 4T1.2HA. After 10 days post tumor injection, mice received an adoptive transfer of 1× 10^6^ activated HA-specific CD8^+^ T cells (48 h post activation) with or without GluR antagonist treatments or saline only (retro-orbitally). 14 days post-tumor injection, mice were euthanized, and the lungs were harvested and used for counting surface metastatic nodules in a blinded way.

#### T cell isolation, activation, and GluR/xCT antagonist treatment

Lymph nodes and spleen cells were pelleted and diluted in MACS solution. According to the manufacturer’s protocol, CD4^+^, CD8^+^ T cells, and tumor-infiltrating lymphocytes (TILs) were isolated using magnetic beads (Miltenyi Biotec, Auburn, CA). For TIL isolation, tumors were digested using a 0.2% type II collagenase (Worthington Biochemical, Lakewood, NJ) dissolved in Dulbecco’s modified Eagle’s medium (DMEM) and incubated for 60 min at 37°C, then minced and homogenized using an 18 G syringe needle and passed through a 40 μm strainer and collected in a 50 mL conical tube using MACS solution. T cells were activated for various times with murine anti-CD3 (17A2) and anti-CD28 (37.51) antibodies at 0.5 μg/mL (Biolegend, San Diego, CA) in complete RPMI media at 37°C, 5% CO_2_ incubator. In the case of alternative activation methods, PMA (50 ng/mL), Ionomycin [Ion] (1μg/mL), and IL-2 (10 ng/mL) were employed. For GluA3, mGluR1, and xCT blocking, NBQX, CPCCOEt (Tocris R&D systems, Minneapolis, MN), and Sulfasalazine (ThermoFisher Scientific) at 60 μM/mL, 75 μM/mL and 0.25 mM,^74^ respectively, were added to cells at the time of activation. For the *in vivo* experiments, CD8^+^ T cells were activated for 48 h, as above, with simultaneous treatment/no treatment with both NBQX and CPCCOEt. After 10 days post-tumor cell injection, Rag2^-/-^ mice received 1 × 10^6^ activated CD8^+^ T cells treated/untreated with GluR antagonists *via* the retro-orbital route.

#### Free glutamate quantification

CD4^+^ and CD8^+^ T cells isolated as described above were activated with anti-CD3 and anti-CD28 antibodies for 24, 36, 48, and 72 h post-activation at 37°C in 5% CO_2_ incubator. Cells were harvested and centrifugated at 1100 rpm for 5 min at 4°C while supernatants were collected and stored at -80°C for glutamate quantification. According to the manufacturer’s instructions, glutamate levels were quantified using a fluorometric Glutamate assay kit (Abcam, cat# ab13883). Briefly, glutamic acid standards were diluted in Assay Buffer plus RPMI media (concentrations varied from 300 to 1 μM) and loaded on 96 well-black flat bottom plates. Samples and blank controls were diluted 1:5 with assay buffer plus RPMI media and plated in triplicate (50 μL/well) on the same plate as standard controls. Next, 50 μL/well of Assay reaction mix with NADP was added to the plate and incubated at room temperature for 30 min. After incubation, fluorescence intensity was measured using the Synergy H1 microplate reader (Biotek) at excitation/emission: 540/590 nm. Glutamic acid concentration was calculated as: Glutamic acid concentration = (glutamic acid in the sample calculated from standard curve / sample volume) x sample dilution factor.

#### Flow cytometry

##### Surface staining

Cells from the activation plate were harvested, transferred to 15 ml conical tubes**,** span for 2000 rpm for 5 min at 4°C**,** and plated onto a 96-well U-bottom plate in blocking buffer containing 1μg/mL anti-CD16/CD32 antibodies in staining buffer (TruStain FcX™, Biolegend) and incubated for 10 min at 4°C. After incubation, antibodies recognizing surface molecules were added to the staining buffer at optimized concentrations for 30 min. Plates were span twice at 2000 rpm for 5 min at 4°C. For the unconjugated antibodies, secondary staining with fluorochrome-conjugated antibodies was added for 30 min and then spanned down as described above. After incubation, cells were analyzed using the flow cytometer Guava EasyCyte HT (Millipore). The following surface antibodies were used. Unconjugated antibodies: anti-glutamate receptor 3 (3B3) (Millipore Sigma), mGluR1, mGluR5, NR1, NR2B (Novus Biologicals R&D systems). Conjugated antibodies (Biolegend): FITC, APC anti-rabbit IgG and anti-mouse IgG, APC anti-mouse CD3, PE, APC, FITC or PerCPCy5.5 anti-mouse CD4 and CD8, PerCPCy5.5 anti-mouse CD11b, APC, FITC anti-mouse CD44, FITC, PE or APC anti-mouse CD25, PerCPCy5.5 anti-mouse CD69.

##### Intracellular staining

Intracellular staining was performed to detect protein phosphorylation (p) and expression of effector molecules. Briefly, after surface staining as described earlier, cells were stained using the Cytofix/Cytoperm kit (BD Biosciences) according to the manufacturer’s instructions. The following antibodies (BD Biosciences) were used: PE anti-mouse IFN-γ, FITC anti-mouse Granzyme B, APC anti-mouse Perforin, unconjugated antibodies pLck, pAKTs, pAKTt, pNFκB p65, pNFAT and pmTOR (Cell signaling) followed by corresponding fluorochrome-conjugated antibodies. Intracellular staining was performed using appropriate dilutions in 1x PermWash buffer for 30 min at 4°C in the dark. After incubation, cells were spun twice at 2000 rpm for 5 min at 4°C following acquisition on the flow cytometer.

##### Acquisition and flow data analysis

Flow samples were acquired on the Guava EasyCyte HT (Millipore) instrument, with 50,000-200,000 cells acquired for each sample. Single color controls determined gates for samples. Unstained and isotype controls were used to ascertain whether any nonspecific binding of antibodies was present. All data were analyzed using FlowJo v10.9 software (TreeStar).

#### Cell viability assay

Cells were stained with 7AAD (ThermoFisher Scientific) according to the manufacturer’s protocol. Cell proliferation was measured using CFSE (Invitrogen CellTrace, ThermoFisher Scientific). In brief, after centrifugation, the cell pellet was diluted in basal RPMI media containing 5 μM CFSE. Cells were stained for 10 min at 37°, centrifuged 3 times in complete RPMI at 2000 rpm for 5 min at 4°C, and incubated at 37°C, with 5% CO_2_ at different time points post-activation.

#### Free glutamate sequestration

To analyze the sequester-free glutamate impact from cell culture supernatant, activated and naive CD8^+^ T cells were incubated with anti-L glutamate 1:100 (ab9490, Abcam) or isotype control (NB810-56910, Novus Biologicals) antibodies for 24, 36, and 48 h post-activation. After incubation, cells were stained for CD25, CD69, and CD44 activation markers. Viability was estimated using 7AAD (ThermoFisher Scientific) as described above.

#### Calcium flux measurement

Activated and naive CD8^+^ T cells were treated with GluR antagonists and incubated at 37°C, 5% CO_2_ for 24 h, as described above. After incubation, cells were harvested from the plate and stained with ratiometric Ca^2+^ dye Fura-2AM (Thermo Fisher) for 15 min. After washing with complete RPMI1640, cells were transferred to a poly-L-lysine coated microscope slide (Fisher Scientific) and spun for 15 min at 1100 rpm, RT. After spinning, cells were visualized using a wide-field microscope (Nikon Eclipse TE 2000E). Fura-2AM fluorescence was monitored using dual excitation wavelengths (340/380 nm) and a single emission wavelength (510 nm). Fluorescence at 340 nm excitation indicates dye bound to Ca^2+^, whereas fluorescence at 380 nm excitation corresponds to free dye. Data are expressed as the ratio of bound: free (340/380 values) Fura 2-AM fluorescence intensities after background subtraction. The ratios of calcium firing were collected for 10 min, at 5-second intervals, where calcium flux values were considered low if the ratio was ≤1.5; moderate, ≥1.5 ≤ 2; and high, ≥ 2.

#### Cytolytic T cell assay

As described above, isolated *Rag2*^-/-^Cln4 CD8^+^ T cells (effectors) were activated and treated with GluR antagonists for 72 h. After 72 h, naive splenocytes (targets) were obtained from WT mice and incubated with 10 μM HA or NP peptide for 2 h. After incubation, cells were spun twice at 2000 rpm for 5 min at 4°C followed by staining of HA-loaded splenocytes with CFSE “high” (5 μM) and NP-loaded (HA^-^) splenocytes with CFSE “low” (0.5 μM) for 10 min. Following centrifugation, the same CFSE “high” and CFSE “low” cell numbers were added to a 96-well plate. CD8^+^ T cells in different ratios were added to each well and incubated at 37°C, 5% CO_2_ for 18 h. Cells were acquired with the flow cytometer, and the frequency of cell lysis was calculated as follows:

$$\%\;Specific\;Lysis=\left(1-\left[Control\;{HA}^{-}{/\;HA}^{+}\right]\;/\;\left[Experiment\;HA^{-}{/\;HA}^{+}\right]\right)\;x\;100$$

#### Confocal microscopy imaging and analysis

HA-specific CD8^+^ T cells were harvested from spleens and lymph nodes of Rag2^-/-^ Cln4 mice and activated/not activated for 48 h with CD3/CD28 antibodies as described above. After 48 h, cells were washed twice with 1xPBS for 5 min, fixed for 10 min in 1% PFA, washed twice with 1x PBS for 5 min, and blocked with 2% FBS for 30 min. Cells were stained with Vβ8.1 (Biolegend) and GluA3 (Millipore-Sigma) antibodies for 30 min, washed twice with 1% FBS in PBS for 5 min followed by incubation with DyLight 405 anti-mouse IgG (Invitrogen), Alexa Fluor 505 anti-rabbit IgG (Invitrogen) and FITC anti-mouse mGluR1a (Novus Biologicals) for 60 min. Cells were washed 2x with 1% FBS in PBS for 5 min, permeabilized with 0.1% Triton-X (Millipore-Sigma) for 15 minutes, stained with eFluor 660 anti-human/mouse pLcK (Tyr505, eBiosciences) for 30 min and washed 2x with 1x PBS plus 1% FBS. Stained cells were mounted in ProLong Diamond Antifade Mountant (Life Technologies) and stored at 4°C in the dark. Images were captured using a Nikon A1R confocal microscope with 60× Plan Apo, 1.4 numerical aperture, with an oil immersion objective, and analyzed using the NIS software. Raw data were exported from the Nikon A1R confocal microscope and were analyzed using Nikon NIS-Elements Advanced Research (USA-NY). To quantify membrane receptor expression, a region of interest (ROI) was drawn around the cytoplasm and nucleus of each cell, using differential interference contrast image (DIC) to define each cell boundary. The mean intensity fluorescence of each receptor (GluA3, mGluR1, and TCR Vβ8.1), as well as the phosphorylated form of Lck (intracellularly), was obtained for each fluorochrome individually, comparing their values between activated and naive CD8^+^ T cells. The colocalization was determined using Pearson coefficients, and values were calculated using the NIS Elements analysis software. For each group, at least 25 cells were counted.

#### Mitochondrial respiration and anaerobic glycolysis evaluation

Mouse lymphocytes were routinely prepared from fresh spleen and lymph nodes of 10 mice pooled together. CD4^+^ or CD8^+^ T cells were isolated *via* a negative selection process using magnetic microbeads as described by the manufacturer (autoMACS; Miltenyi Biotec, Cologne, Germany). Metabolic analysis was performed using the Extracellular Flux Analyzer XF^96^ (Seahorse Agilent Technologies). Briefly, 2.5 x 10^5^ cells/well were seeded onto Poly-D-Lysine treated, washed, and dried Agilent Seahorse 96-well microplate. At least 5 wells/conditions were used as replicates. Lymphocyte growth media (RPMI media with 200mM L-glutamine, 1mM Sodium pyruvate, HEPES (5 mL), 0.1% 2-Mercaptoethanol, NEAA (5 mL), and 10% FBS) was used for plating, culturing, and activating T cells. For activation, cells were treated with CD3/CD28 antibodies (1 μg/ml) for 48 h. CPCCOEt, a GluR1 inhibitor, was added at the time of activation at 75 μM concentration. Mitochondrial stress and glycolytic parameters were measured on the same plate in two separate assays using oxygen consumption rate (OCR) (pmoles/min) and extracellular acidification rate (ECAR) (mpH/min), respectively, with the use of real-time injections. For mitochondrial stress assay (Seahorse Agilent Technologies), XF basic assay media (RPMI, pH 7.4) supplemented 1 mM sodium pyruvate, 2 mM L-glutamine, and 25 mM Glucose and subsequent injections of oligomycin (1.0 μM), carbonyl cyanide-*4*-(trifluoromethoxy) phenylhydrazone (FCCP; 2 μM) and rotenone plus antimycin (both 0.5 μM) were used. For glycolysis, XF assay (Seahorse Agilent Technologies) media (RPMI, pH 7.4) with 1 mM glutamine and injections of glucose (11.1 mM), oligomycin (0.75 μM) and 2-deoxy-d-glucose (100 mM) were used. Cell/protein load was normalized using the SRB kit (Abcam). Seahorse analysis was performed 48 h post-incubation. Calculations of individual metabolic parameters were done using Wave 2.6.3 software per the manufacturer’s instructions (Seahorse Agilent Technologies).

### Quantification and statistical analysis

All experiments were conducted independently in three independent replicates. GraphPad Prism 9.5.2 software (GraphPad Software, Inc., San Diego, CA, USA) was used for graphical representation and statistical analysis. Normality, homogeneity of variance, and data independence were determined before every analysis. All the parametric variables were analyzed with a two-tailed unpaired *t*-test or two-way ANOVA with Bonferroni correction for multiple comparison tests in all the experiments. Data were presented as mean ± SEM, and the *p* value < 0.05 was considered statistically significant. The statistical details of the experiments are also presented in the figure legends.

## Supplemental information

Document S1. Figures S1–S7 and Table S1

Figure360. An author presentation of Figure 1

Figure360. An author presentation of Figure 2

Figure360. An author presentation of Figure 3

Figure360. An author presentation of Figure 4

Figure360. An author presentation of Figure 5

Figure360. An author presentation of Figure 6

Figure360. An author presentation of Figure 7

Video abstract
