# Supplementary material for: Glutamate receptor–T cell receptor signaling potentiates full CD8+ T cell activation and effector function in tumor immunity
Source: iScience. 2025 Aug 11;28(10):112772. doi: 10.1016/j.isci.2025.112772 (PMC12529521; doi:10.1016/j.isci.2025.112772)
Supplement: Document S1. Figures S1–S7 and Table S1 [file mmc1.pdf]

iScience, Volume *28*

Supplemental information

Glutamate receptor–T cell receptor signaling potentiates full CD8$^+$ T cell activation and effector function in tumor immunity

Maria Teresa P. de Aquino, Thomas W. Hodo, Salvador González Ochoa, Roman V. Uzhachenko, Muna A. Mohammed, J. Shawn Goodwin, Thanigaivelan Kanagasabai, Alla V. Ivanova, and Anil Shanker

Table S1. Flow cytometry antibodies, related to STAR Methods.

**REAGENT or RESOURCE**	**SOURCE**	**IDENTIFIER**
**Antibodies**		
TruStain FcX™	Biolegend®	(Cat# 101320, RRID: AB_1574975)
Anti-Glutamate Receptor 3 Antibody, clone 3B3	Sigma-Aldrich	(Cat# MAB5416, RRID: AB_2113897)
Anti-mGluR1	Novus Biologicals R&D systems	(Cat# NBP1-50203, RRID: AB_10012403)
Anti-mGluR5	Novus Biologicals R&D systems	(Cat# MAB4514, RRID: AB_2232846)
Anti-NR1	Novus Biologicals R&D systems	(Cat# PPS083, RRID: AB_2112007)
Anti-NR2B [pTyr1336]	Novus Biologicals R&D systems	(Cat# PPS057, RRID: AB_2112917)
FITC Donkey anti-rabbit IgG	Biolegend®	(Cat# 406403, RRID: AB_893531)
APC anti-mouse IgG1 Antibody	Biolegend®	(Cat# 406610, RRID: AB_10696420)
FITC anti-mouse IgG1 Antibody	Biolegend®	(Cat# 406606, RRID: AB_493293)
APC anti-mouse CD3 Antibody	Biolegend®	(Cat# 100236, RRID: AB_2561456)
PerCP/Cyanine5.5 anti-mouse/human CD11b Antibody	Biolegend®	(Cat# 101228, RRID: AB_893232)
PerCP/Cyanine5.5 anti-mouse CD4 Antibody	Biolegend®	(Cat# 100434, RRID: AB_893324)
PE anti-mouse CD4 Antibody	Biolegend®	(Cat# 100408, RRID: AB_312693)
APC anti-mouse CD4 Antibody	Biolegend®	(Cat# 100412, RRID: AB_312697)
FITC anti-mouse CD4 Antibody	Biolegend®	(Cat# 100406, RRID: AB_312691)
PerCP/Cyanine5.5 anti-mouse CD8 Antibody	Biolegend®	(Cat# 100734, RRID: AB_2075238)
PE anti-mouse CD8a Antibody	Biolegend®	(Cat# 100708, RRID: AB_312747)
APC anti-mouse CD8a Antibody	Biolegend®	(Cat# 100712, RRID: AB_312751)
FITC anti-mouse CD8a Antibody	Biolegend®	(Cat# 100706, RRID: AB_312745)
APC anti-mouse CD44 Antibody	Biolegend®	(Cat# 103012, RRID: AB_312963)
FITC anti-mouse CD44 Antibody	Biolegend®	(Cat# 103006, RRID: AB_312957)
FITC anti-mouse CD25 Antibody	Biolegend®	(Cat# 102006, RRID: AB_312855)
PE anti-mouse CD25 Antibody	Biolegend®	(Cat# 102008, RRID: AB_312857)
APC anti-mouse CD25 Antibody	Biolegend®	(Cat# 101910, RRID: AB_2280288)
PE/Cyanine7 anti-mouse CD62L Antibody	Biolegend®	(Cat# 104418, RRID:AB_313103)

APC anti-mouse TIGIT (Vstm3) Antibody	Biolegend®	(Cat# 156106, RRID:AB_2750515)
CTLA-4 Monoclonal Antibody (1B8), FITC	Thermo fisher	(Cat# HMCD15201, RRID:AB_2536593)
PerCP/Cyanine5.5 anti-mouse CD69 Antibody	Biolegend®	(Cat# 104522, RRID: AB_2260065)
Anti-Lck, phospho (Tyr505) Antibody, Unconjugated	Cell signaling	(Cat# 2751, RRID: AB_330446)
Phospho-Akt (Thr308) (C31E5E) Rabbit mAb	Cell signaling	(Cat# 2965, RRID: AB_2255933)
Unconjugated anti-rabbit AKT	Cell signaling	(Cat# 4685, RRID: AB_2225340)
Unconjugated anti-rabbit NFκBp65	Cell signaling	(Cat# 8242, RRID: AB_10859369)
Unconjugated NFAT1 (phospho Ser54)	GeneTex	(Cat# GTX25246, RRID: AB_380487)
Unconjugated SLC1A5 Antibody	Novus Biologicals R&D systems	(Cat# NBP1-89327, RRID: AB_11024237)
Anti-L Glutamate antibody	Abcam	(Cat# ab9440, RRID: AB_307256)
Rabbit IgG Isotype Control	Novus Biologicals R&D systems	(Cat# NB810-56910, RRID: AB_844243)
Purified anti-mouse TCR Vβ8.1, 8.2	Biolegend®	(Cat# 118402, RRID: AB_1027707)
Goat anti-Mouse IgG (H+L) Secondary Antibody, DyLight™ 405	Thermo fisher	(Cat# 35501BID, RRID: AB_2533209)
Goat anti-Rabbit IgG (H+L) Cross-Adsorbed Secondary Antibody, Alexa Fluor™ 555	Thermo fisher	(Cat# A-21428, RRID: AB_2535849)
mGluR1 Antibody [FITC]	Novus Biologicals R&D systems	(Cat# NB100-93555F, RRID: AB_3174610)
xCT Antibody [PE]	Novus Biologicals R&D systems	(Cat# NB300-318PE, RRID: AB_3187394)
SLC1A2 / EAAT2 / GLT-1 Antibody [APC]	LS Bio	(Cat# LS-C516967-100, RRID: AB_3361348)
GLS Antibody [PE]	LS Bio	(Cat# LS-C728142-100, RRID: AB_3361703)
Phospho-LCK (Tyr505) Monoclonal Antibody (SRRCHA)	eBioscience™	(Cat# 50-9076-42, RRID: AB_2574315)
AlexaFluor 555 goat anti-rat IgG	Invitrogen	(Cat# A-21434, RRID: AB_2535855)
Goat Anti-Mo IgG DyLight 405	Invitrogen	(Cat# 35500BID, RRID: AB_2533208)

Figure S1

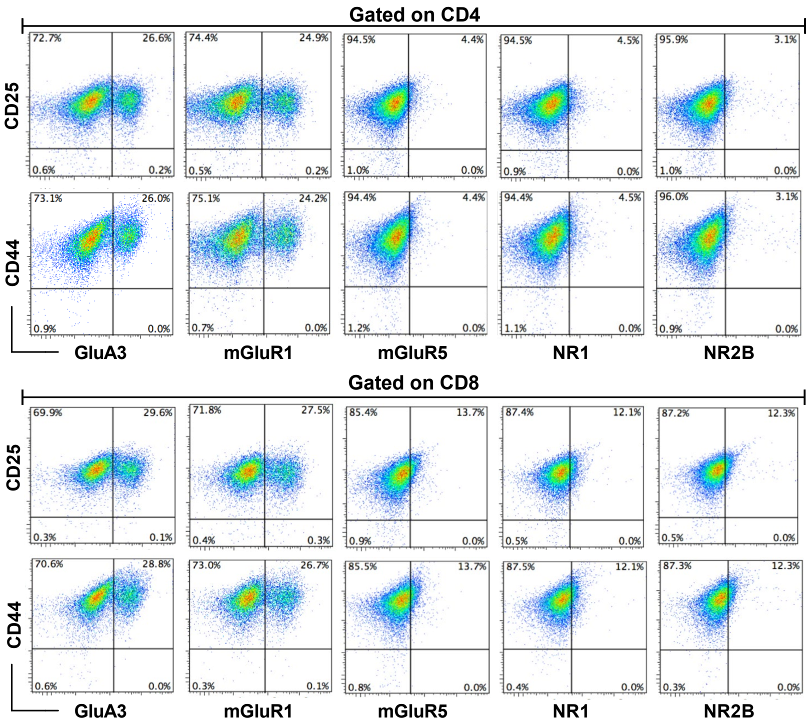

Figure S1. The upregulation of GluRs is correlated with heightened activation in tumor-infiltrating lymphocytes. Related to Figure 1. Representative dot plots show GluRs vs. CD25 and CD44 expression on gated $CD4^+$ and $CD8^+$ tumor-infiltrating T-cells. These dot plots data are representative of 3 independent experiments.

Figure S2

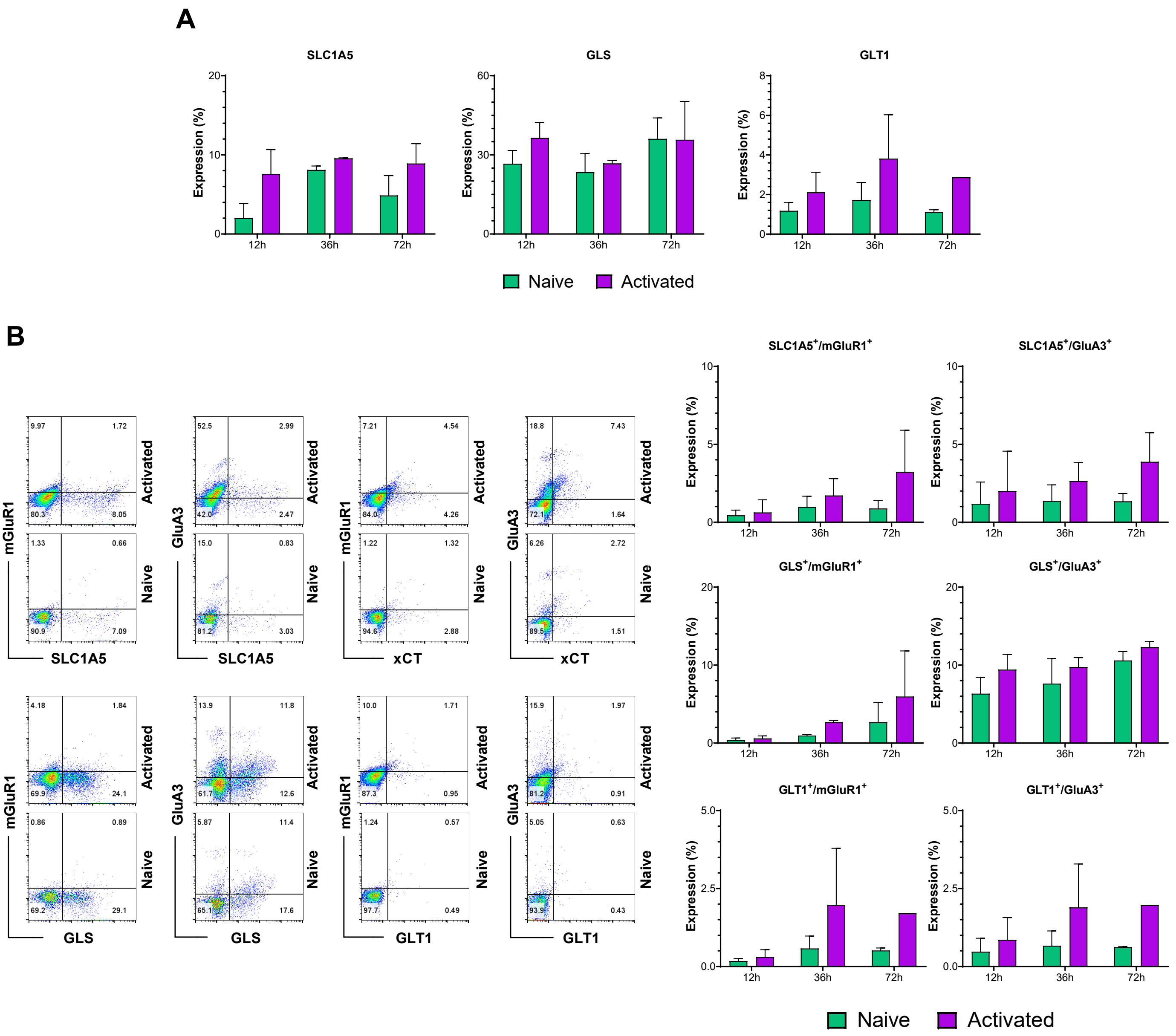

Figure S2. The regulation of alternative glutamate transporters expression is not associated with CD8^{+} T-cell activation. Related to Figure 2. A. Bar graph depicting non-significant changes in glutamate transporters at 12-, 36-, and 72-h post-activation. Bar graphs represent the Mean ± SEM (n= 3). **B.** Visualization analysis of glutamate transporter and GluR coexpression using dot plots and bar graphs. These dot plots data are representative of 3 independent experiments. Bar graphs represent the Mean ± SEM (n= 3).

Figure S3

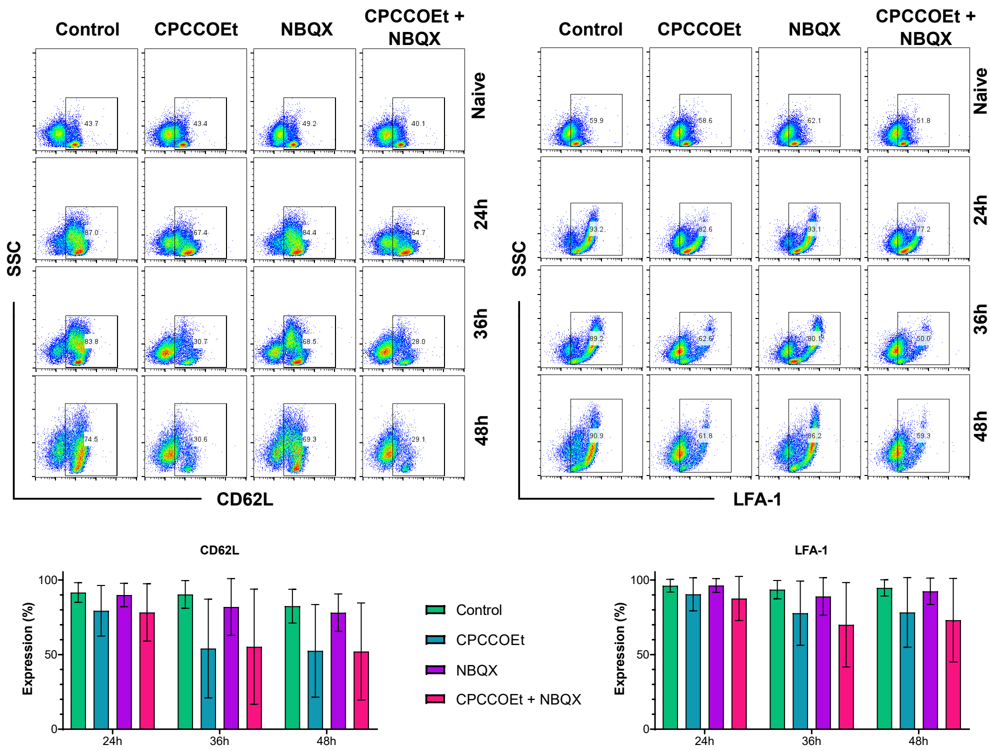

Figure S3. GluA3 and mGluR1 inhibitors do not affect T-cell memory and adhesion molecules. Related to Figure 3. Evaluation of the impact of GluRs antagonist on the expression of CD62L and LFA-1 in activated $CD8^+$ T-cells at 24, 36, and 48h post-activation. These dot plots data are representative of 3 independent experiments. Bar graphs represent the Mean ± SEM (n= 2).

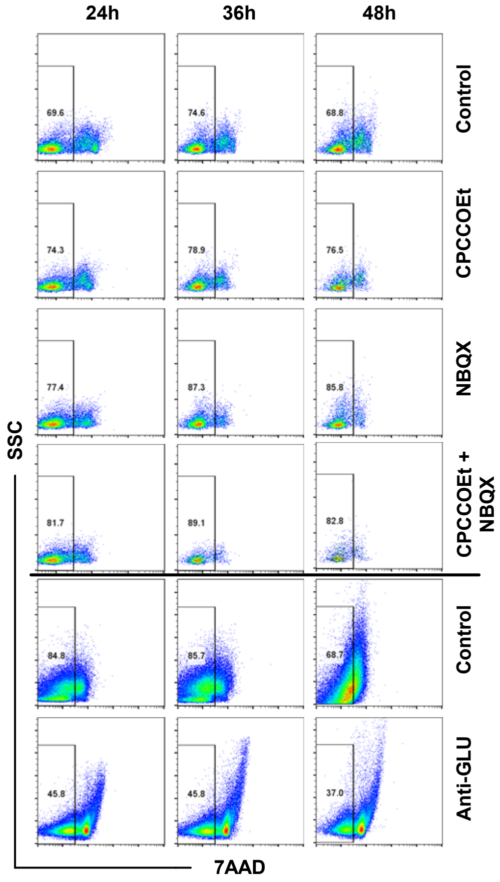

Figure S4. Inhibition of GluRs does not affect CD8$^+$ T-cell viability during activation. Related to Figure 3. Dot blots depict the assessment of the cell viability of isolated CD8$^+$T-cells treated with GluR antagonists or anti-glutamate antibodies at different time points after activation. These dot plots data are representative of 3 independent experiments.

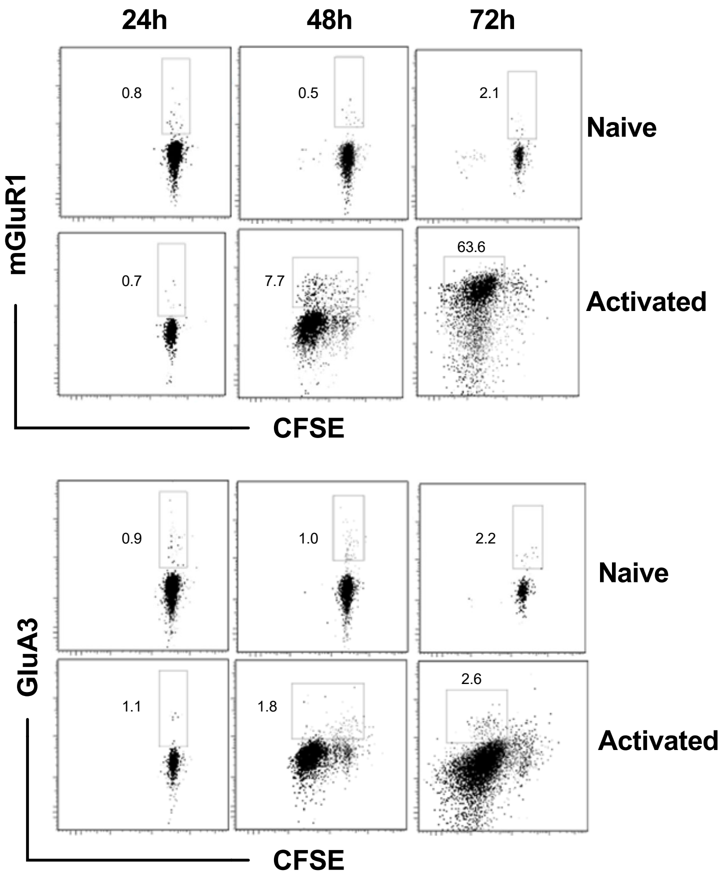

Figure S5. The upregulation of mGLuR1 expression correlates with an increase in the proliferation capacity of CD8 T lymphocytes. Related to Figure 6. Dot plots displaying the expression of mGluR1 and iGluR3 on isolated proliferative CD8$^+$ T-cells measured after 24, 48, and 72 h of *in-vitro* activation. The upper panels show the frequency of mGluR1$^+$CFSE$^+$ 7AAD$^-$ cells on activated and naive CD8$^+$ T-cells, while the lower panels show the frequency of iGluR3$^+$CFSE$^+$ 7AAD$^-$ cells on activated and naive CD8$^+$ T-cells.

Figure S6

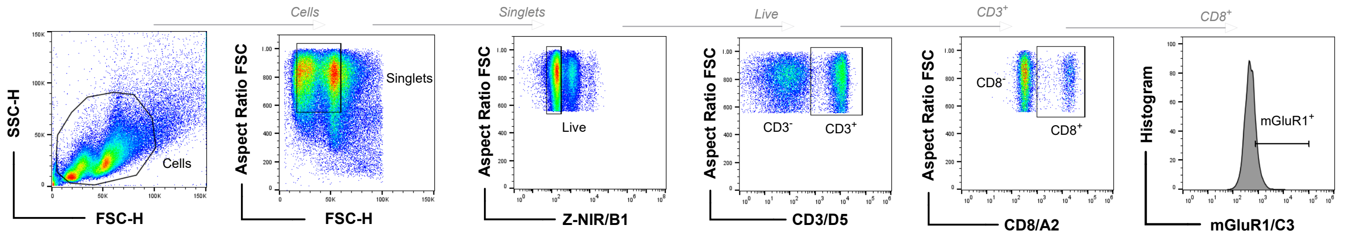

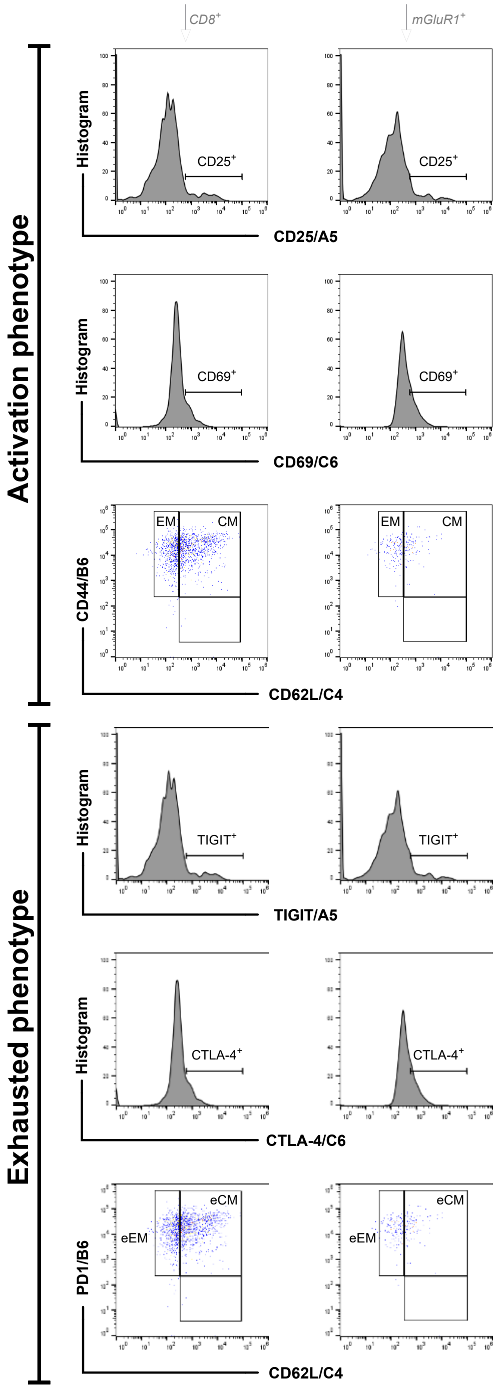

Figure S6. Gating strategy for analyzing activating and exhausted markers on mGluR1$^{+/-}$ CD8^{+} T-cells from TDLN and TILs. Flow cytometry gating was performed to identify mGluR1 expression and characterize CD8^{+} T cell states. [Cells] were gated for [singlets], [live cells], and [CD3^{+}] [CD8^{+}] T cells, followed by the analysis of [mGluR1^{+}] expression. Activation markers (CD25, CD69) were assessed on mGluR1^{+}CD8^{+} T cells. Memory subsets were distinguished as central memory (T_{CM}: CD44^{+}CD62L^{+}) and effector memory (T_{EM}: CD44^{+}CD62L^{-}), while exhaustion was evaluated using TIGIT^{+}, CTLA-4^{+}, exhausted central memory (eT_{CM}: PD1^{+}CD62L^{+}), and exhausted effector memory (eT_{EM}: PD1^{+}CD62L^{-}) gates.

Figure S7

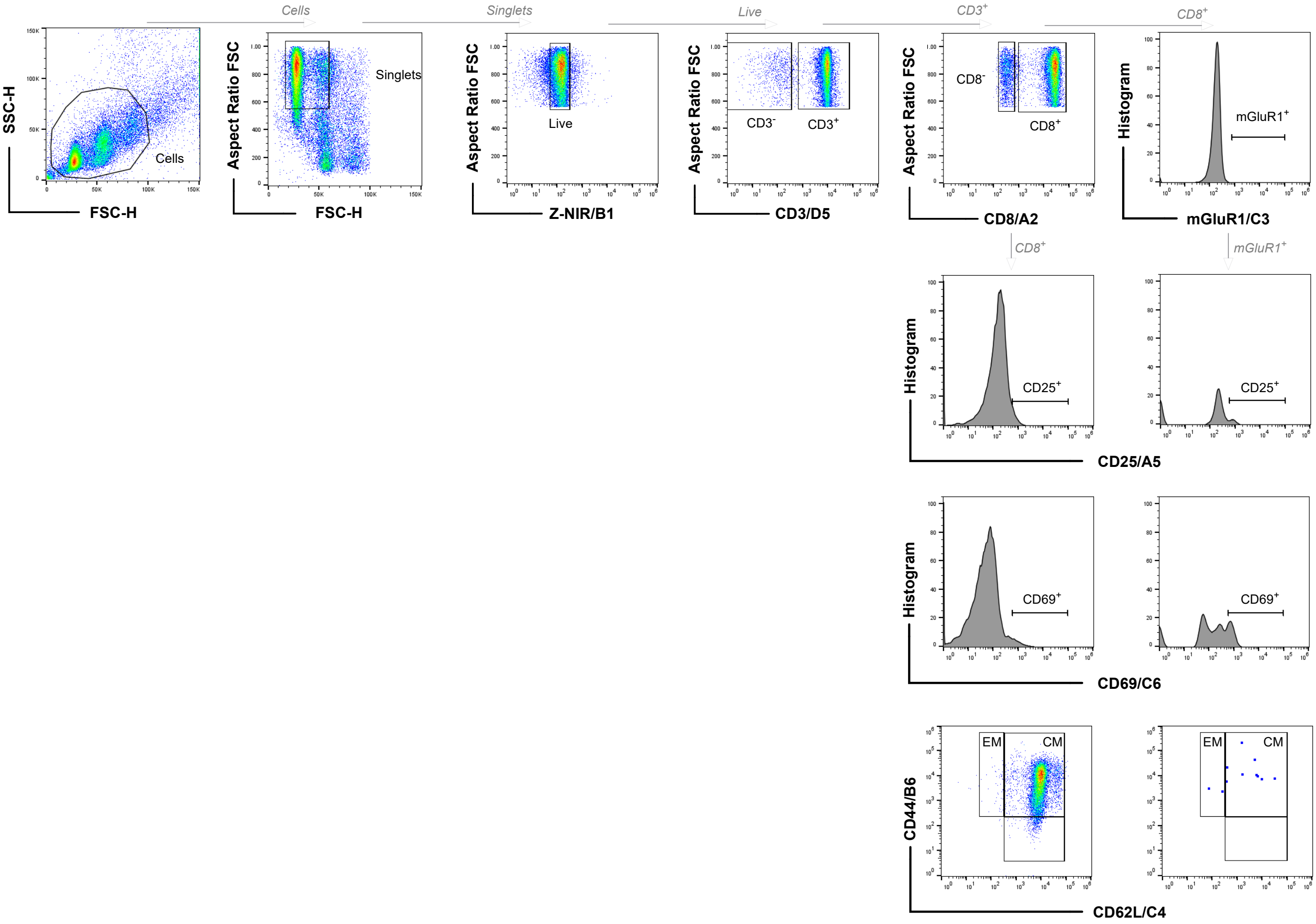

Figure S7. Gating strategy for analyzing activating markers on mGluR1$^{+/-}$ CD8^{+} T-cells treated/ not treated with mGluR1 and xCT inhibitors. Flow cytometry gating was performed to identify mGluR1 expression and characterize CD8^{+} T cell states. [Cells] were gated for [singlets], [live cells], and [CD3^{+}] [CD8^{+}] T cells, followed by the analysis of [mGluR1^{+}] expression. Activation markers (CD25, CD69) were assessed on mGluR1^{+}CD8^{+} T cells. Memory subsets were distinguished as central memory (T_{CM}: CD44^{+}CD62L^{+}) and effector memory (T_{EM}: CD44^{+}CD62L^{-}) gates.
